# Fair allocation strategies for opioid settlements

**DOI:** 10.1007/s10729-025-09716-8

**Published:** 2025-08-07

**Authors:** Qiushi Chen, Robert Newton, Paul Griffin

**Affiliations:** 1 Harold & Inge Marcus Department of Industrial and Manufacturing Engineering, The Pennsylvania State University, University Park, Pennsylvania, 16802 USA; 2 Consortium on Substance Use and Addiction, Social Science Research Institute, The Pennsylvania State University, University Park, Pennsylvania, 16802 USA

**Keywords:** Fairness, Resource allocation, Opioid epidemic, Frontier, Disparity, Interpretability, Operations research

## Abstract

Multi-billion-dollar opioid settlement agreements have been reached with pharmaceutical manufacturers and distributors to address their liability in contributing to the opioid epidemic in the United States. These agreements stipulate that within the state, the settlement funds must be directly allocated to local government (e.g., counties) and used for abatement activities to remediate the harm of the opioid epidemic in communities. This naturally leads to an important question of how the funds should be distributed to meet the diverse needs of the counties consistently across all counties to be deemed fair. Although there exist various definitions of fairness in the literature, it remains unclear how to empirically quantify the fairness of settlement allocation based on data, which is crucial for developing evidence-based allocation policies. To fill this gap, we define two allocation fairness measures, deviation and maximum regret, and formulate the fair settlement allocation as convex optimization problems. To further enhance the interpretability of the allocation policies, we restrict the allocation to a weighted sum of the given empirical metrics. We apply our analytical framework in a case study of the settlement allocation in Pennsylvania using real-world empirical metrics. We identify the frontiers of the non-dominated allocation policies between min-deviation and minimax-regret allocations, which dominate all alpha fairness-based and formula-based allocation policies. All allocation policies show lower fairness (with higher deviation or maximum regret) in counties that are rural, low-income, and with lower-ranking health factors. The price of interpretability is more significant in terms of maximum regret compared with deviation.

**Supplementary Information:**

The online version contains supplementary material available at 10.1007/s10729-025-09716-8.

## Highlights

- We develop an analytical framework for fair allocation of the opioid settlement, which defines two context-specific allocation fairness measures, namely, *deviation* and *maximum regret*, and formulates the fair allocation decision questions as convex optimization problems.
- We present the *deviation-regret frontier* showing the trade-off between the two fairness measures and the boundary beyond which the two fairness measures cannot be further improved simultaneously by any other feasible allocation strategy.
- We examine the disparities in allocation fairness across counties by several social determinants of health, and find that counties in rural areas, with low income, and with lower-ranking health factors may be disadvantaged with lower allocation fairness measures across all allocation strategies.
- Incorporating interpretability constraints affects the maximum regret more than the deviation and moves the frontier further away from the origin, demonstrating the trade-off between interpretability and fairness when choosing allocation strategies.

**Supplementary Information:**

The online version contains supplementary material available at 10.1007/s10729-025-09716-8.

## Introduction

More than a million Americans have died from drug overdoses since 1999 [1]. The collision of the opioid epidemic with the COVID-19 pandemic further escalated the drug overdose crisis. Annual overdose deaths in the United States (US) reached an all-time high in 2021 at 107,000 deaths [2], meaning that at least one drug overdose death occurred every five minutes. As overdose deaths involving synthetic opioids such as fentanyl and stimulants including cocaine continue to rise, the opioid epidemic has shifted from a crisis of prescription opioids and heroin to synthetic opioids with adulterants and beyond opioids, now driven by stimulants and polysubstance use in the “fourth wave” [3]. Beyond the devastating fatal burden on society, the opioid epidemic had deleterious impacts on healthcare, criminal justice, and productivity [4], with an estimated cost to the US of $1.5 trillion in 2020 [5].

### Opioid settlement and settlement allocation

The burden of addressing the impacts of the opioid epidemic on affected communities has largely fallen on the public sector. Government entities from the federal to the municipal level have borne the brunt of the costs associated with addressing both the short- and long-term needs of those affected by the opioid crisis. As it is generally agreed that the epidemic was fueled by the actions of prescription opioid manufacturers and distributors in the late 1990s [6, 7], states and municipalities began attempting to hold these companies responsible, filing lawsuits in the early 2000s [8]. Since 2014, more than 3,000 civil lawsuits have been filed by various jurisdictions across the country against opioid pharmaceutical manufacturers, distributors, and retailers. The concerted efforts of attorneys general across the nation ultimately led to a historic lawsuit—in 2021, 46 states reached a landmark settlement agreement of $26 billion over an 18-year period with three pharmaceutical distributors (AmerisourceBergen, Cardinal Health, and McKesson) [9] and one prescription opioid manufacturer (Janssen) [10]. While this is the largest opioid-related settlement to date, it is by no means the last. Recently, we have seen additional settlements with Teva [11], Allergan [12], and several pharmacy retailers like CVS and Walgreens [13, 14].

These recent and pending legal settlements with opioid distributors and manufacturers lead directly to a discussion of how to disburse settlement funds to states. Unlike the 1998 Master Settlement Agreement with over 45 tobacco companies [15, 16], for which one critique was how little of the money was held at the local level and hence did not get spent within communities [17], the 2021 opioid settlement agreement stipulates that at least 85% of the funds go directly to participating states and subdivisions (e.g., counties and municipalities) and must be used for abatement activities to remediate the harm of the opioid epidemic in communities. However, there is no standard approach to allocating the settlement funds across counties within the state. Although a formula was used for distributing the national settlement across states based on four state-level empirical measures, using the exact same formula is not always feasible at the county level due to the lack of county-level data for some of these measures. Different states have documented different approaches of distributing funds to local governments [14], such as by population (e.g., Kansas [18]) or using formulas with modified metrics or weights (e.g., Tennessee [19] and Maryland [20]).

While the opioid crisis has widespread impact across the nation, not all communities have been affected in the same way. It is imperative, therefore, to develop a fair allocation strategy to help communities remedy the harms and make meaningful progress in addressing this tragedy by utilizing the settlement funds. Communities have different root causes for substance misuse and thus have diverse needs for mitigation. The breadth of effects resulting from opioid and substance misuse makes quantifying community needs a unique challenge. In addition, the concept of fairness, despite its importance, has no generally accepted standard definition or formulation for quantification and interpretation. A recent practical guide to formulating fairness [21] summarizes a wide range of fairness definitions based on inequality, outcomes for the disadvantaged, and combinations of fairness with efficiency, which can be used to formulate resource allocation problems with fairness objectives as computationally tractable optimization models. While serving as valuable references, these fairness measures do not result in immediate solutions to our fair allocation problem for the opioid settlement, as the gaps in how to incorporate empirical data in the formulation of fairness for a specific problem context remain to be bridged.

One unique aspect of fairness in the intrastate opioid settlement allocation problem is that the state’s goal is to propose a fair allocation policy to incentivize all political subdivisions (e.g., counties) to participate in the joint settlement. This is because the settlement agreement stipulates that the full amount of the incentive payments in the settlement funds is earned by resolving existing and barring future claims by all subdivisions against these pharmaceutical companies [22] (see more details in the settlement agreements [9, 10]). In other words, full participation by local counties is required to receive maximum settlement funds [23]. Counties are given a limited time window to decide whether they see their allocated amount as “fair” and reasonable and hence accept their allocation that is risk-free by signing the agreement, or pursue lawsuits on their own for a possibly higher settlement amount but also at a higher risk. As the acceptability of the allocation is somewhat subjective for each county in this case, the central decision maker’s strategy to have all counties’ buy-in in a timely manner is to develop a reasonable allocation plan using transparent data and an impartial approach to ensure that all counties are relatively well provided with resources to address their needs. Thus, this specific problem context warrants additional careful thoughts in the design of fairness measures for the allocation policy of opioid settlement funds.

### Overview

We formulate the fair settlement allocation problem as an optimization model: Given a set of empirical metrics that represent the heterogeneous burden of the opioid epidemic and the level of need for each county, what is the best way to distribute a fixed total amount (100%) of settlement funds to each county to maximize the overall allocation fairness from a central decision maker’s viewpoint (e.g., state attorney general)? Specifically, we propose two *allocation fairness* criteria based on empirical data in the context of the opioid settlement allocation:

1. *Minimizing deviation* (“min-deviation”): It views existing empirical metrics for a county as multiple observations of the true need of that county, aiming to align the allocation amount with these metrics as close as possible by minimizing their “distances.”
2. *Minimizing maximum regret* (“minimax-regret”): We quantify the regret of a county as the relative difference between the allocated percentage and the maximum percentage that the county could justify by using the highest empirical metric. Acknowledging that not all counties will be allocated by their highest empirical metric, the minimax-regret criterion aims to make the highest regret as low as possible, so that the most disadvantaged county would not feel substantially worse off than the rest, which would eventually help achieve the most buy-in from all counties for signing on the agreement.

In addition, we also include *alpha fairness* in our formulation, a commonly used measure in the existing fairness literature that balances between efficiency and equity. Using real-world empirical metrics data, we perform a case study in the setting of the settlement allocation in Pennsylvania. We numerically solve the optimal allocation policies under different fairness criteria, compare allocation policies and discuss practical implications by different fairness formulations, and further investigate the disparity and interpretability of allocation policies that are critical considerations in the opioid settlement allocation.

Our main contributions in this study are three-fold. First, we propose an evidence-based analytical framework for the fair allocation in the problem context of opioid settlement allocation from a central decision maker’s (e.g., state government) perspective. Most of the existing fairness measures used for optimizing resource allocations in generic settings are based on the distribution of utilities across multiple entities. However, it remains unclear as to how the concept of fairness can be operationalized to guide the allocation of opioid settlement funds given a set of empirical data. We develop two fairness measures, *deviation* and *maximum regret*, that are motivated and rationalized based on the general equity principle of *allocation according to need* to quantify how well the allocated shares are aligned with the needs consistently across all counties using empirical data, resulting in allocation policies that are *evidence-based*.

Second, we formulate the class of interpretable allocation policies and construct the *deviation-regret frontier* to help decision makers better compare and understand the values of allocation policies with various practical considerations. Specifically, to enhance the interpretability of the allocation policy created from the *black-box* optimization model, we restrict the allocation policy to be parameterized as a weighted sum of empirical metrics, a simple mathematical form that is appealing to decision makers (as the allocation formula used in several practical cases) due to its simplicity and transparency. We further quantify the *price of interpretability* to show the trade-offs between solution complexity and fairness outcome, which can be used to facilitate communication with community stakeholders. In addition, we create the *deviation-regret* frontier showing the trade-off between the two proposed fairness measures. The decision maker can set an acceptable value of one of the measures (e.g., maximum regret) and find the policy that optimizes the other (e.g., deviation). The frontier also visualizes the boundary for all feasible allocation policies in the fairness space, which can serve as a useful reference for decision makers to assess the potential room for improvement in fairness for any given allocation policy of interest.

Lastly, we address disparities resulting from allocation policies, which are not directly accounted for by the fairness measures themselves but are also important considerations in practice. To examine such disparities, we assess the differences in the fairness measure between subgroups of counties categorized by social determinant of health factors and then compare such differences across allocation policies.

The remainder of this paper is organized as follows. We first review the literature related to fairness and resource allocation in Section 2. In Section 3, we describe a real-world case of the opioid settlement allocation in Pennsylvania to motivate further analysis using modeling approaches to address the fair allocation problem. In Section 4, we present optimization models of fair allocation under different definitions of fairness in the context of opioid settlement. In Section 5, we present a numerical case study using real-world empirical data from Pennsylvania, and discuss the results and their implications from the computational analysis. Lastly, we give concluding remarks and discuss future research in Section 6.

## Literature review

It may seem intuitive to allocate resources with the goal of maximizing overall effects or benefits, but in the case of public funds, the effects of such allocations are often indirect or difficult to measure, as they are focused on social outcomes such as poverty, community health, or productivity [24, 25]. When allocating resources to the public, multiple indicators should be used to measure their impact and guide planning, rather than focusing on a single indicator, which could yield unintended consequences in other dimensions at the expense of overall societal welfare [26–28]. However, allocating funds according to multiple and potentially conflicting attributes is difficult to optimize, as modeling interactions or synergies of various expenditures in a multi-criteria model requires a great degree of computational effort [25]. As such, many models account for a single attribute of the community such as population size [29–31]. Decision support tools like Analytical Hierarchy Process [32] and outranking [33] provide decision makers with options to prioritize multiple attributes, but leave it to the decision maker to choose among a set of alternatives. Goal programming presents an optimal allocation across a set of possible (often binary) expenditures by minimizing deviations, but these deviations are commonly based on expert assessments and do not necessarily consider fairness [34].

In the allocation of public resources, fairness and equity must be considered, although they are not often well defined [25, 35–37]. Fairness and equity are often conflated, as their definitions are related but not agreed upon [38]. Fairness can be understood as the perceived acceptability of what one party receives relative to another party [39]. Equity is often concerned with satisfying the relative needs of a group or community, defined by demographics or geography [30], where these needs are satisfied through the allocation of scarce resources. For fair division of resources, envy-freeness is one of the most prominent notions and criteria [40], which means that each individual agent’s utility of what they receive is at least as high as their utility of what others are allocated (i.e., in their eyes). Simply put, no agent prefers the allocation of another. In the economics literature, envy-free allocations are also held as efficient [41, 42].

A recent survey by Chen and Hooker [21] reviews a broad range of fairness criteria for utility distribution that can be used to maximize the social welfare function for allocating resources. It categorizes the fairness metrics into several major groups as inequality measures, the utility for the disadvantaged group, and the combination of efficiency (total utility) and fairness. Inequality measures (e.g., range, deviations, coefficient of variation, Gini coefficient, Hoover index), for quantifying the spread in the distribution of utilities, have also been used for generic outcomes beyond utility in applications of location questions [43], communication networks [44], scheduling and transportation [45]. A review of equity measures in facility location models [31] provides a general framework, which consists of three dimensions in reference distribution, scale, and metric, for defining equity measures. The maximin criterion, popularized by Rawls’s [46] work in social contract theory, maximizes the average utility of a group by benefiting the least advantaged members of that group. To combine efficiency and fairness, alpha fairness provides a continuum from strictly utilitarian ($\alpha =0$) to maximin ($\alpha \rightarrow \infty$) [21]. Maximizing alpha fairness with $\alpha = 1$, called *proportional fairness*, yields the solution known as the Nash bargaining solution [47], which is further expanded by Kalai-Smorodinsky’s bargaining solution [48] that allots the parties the largest possible fraction of their potential utility while observing fairness by equalizing that fraction. Notably, alpha fairness is closely related to the Atkinson social welfare index [49], which measures how much social utility can be gained by redistributing income, or more simply inequality. Given the selected $\alpha$ value, maximizing alpha fairness and minimizing the Atkinson Index are reduced to the same mathematical representation. The parameter $\alpha$ in alpha fairness becomes the same as the “inequality aversion parameter” following the definition of the Atkinson Index, which has been estimated empirically in several social settings [50, 51].

A related issue to fairness in the context of decision making is the notion of regret. Previous work has shown that decision makers will not always maximize expected utility in the face of uncertainty, and much of this behavior can be explained by the alternative objective of minimizing decision regret [52, 53]. Regret exists when a decision to deviate from a benchmark or a target turns out to perform poorly [54, 55]. Aggregating individual positions to minimize group regret can also maximize group satisfaction [26]. In the case of allocating funds across multiple sub-state entities, we can define regret as the deviation from a benchmark from below.

Several researchers have developed fair allocation mechanisms that share some similarities to our work. Wei et al. [56] study the problem of a funding agency providing resources to several service agencies to generate societal benefits from the allocation. They consider equity of the allocation in terms of the outcomes of the resulting societal impact and show how to modify the allocation to balance the outcomes. Arnosti and Shi [57] consider the problem of allocating housing vouchers to low-income families in order to maximize the social welfare through a lottery mechanism. Zaric et al. [58] consider a hierarchical resource allocation problem for HIV prevention programs where a central agency funds regional decision makers that then fund local programs. They study the trade-off of proportional funding based on HIV incidence to efficient funding based on maximizing HIV infections averted. They further consider program incentives by the higher-level entities that would promote efficient allocations at the lower level. Lasry et al. [59] consider a similar framework for developing countries, and define optimal allocations by minimizing the number of new infections while accounting for the equity in terms of the number of HIV cases in each local region. They show the importance of focusing on optimization at the lower-level allocation decisions. Wang et al. [60] study the allocation of voting machines as a public resource with a bi-objective optimization model involving two conflicting objectives of efficiency (measured by average waiting time) and equity (measured by the range of waiting time). They employ an epsilon-constraint solution method to solve for non-dominated solutions and construct the efficiency-equity frontier to quantify the trade-off between efficiency and equity.

Our problem differs from past research in several important ways. The notion of “fairness” in our problem should be interpreted under the specific problem context of settlement allocation, which is based on how well the allocated resources are aligned with community needs, following the equity principle of *allocation according to need*, rather than measuring the outcomes of the allocated resources (e.g., population health outcomes or generic utility functions) and then maximizing the total social welfare. In most other modeling studies for fair resource allocation, the outcomes of allocated resources could oftentimes be directly computed based on their problem settings. However, in our problem, it remains unclear how to measure the alignment between settlement allocation and the communities’ needs based on a given set of empirical data representing the damages of the opioid crisis to communities. Thus, it warrants the development of new formulations and analytical approaches to address the fair settlement allocation problem.

## A motivating problem: the opioid settlement in Pennsylvania

As a result of the $26 billion national settlement reached between attorney generals from 46 states and four opioid manufacturers and distributors in 2021 [61], Pennsylvania will receive $1.07 billion in settlement funds over the next 18 years. Overseen by an appointed Board of Trustees, Pennsylvania must use these settlement funds for abatement activities, addressing the impacts of the opioid epidemic [62]. Pennsylvania directly allocated 70% of its total funds across its 67 counties, 15% to local litigating entities, and reserved the remaining 15% for the state government. State and local government entities can choose to implement abatement activities from a wide range of approved intervention strategies to address misuse of opioids, as well as other substances like stimulants [61].

At its outset, however, the settlement did not guarantee the full amount of calculated funds allocated to each state. Settlement funds consist of base and incentive payments. To receive the full amount of the incentive payment, participating states needed to ensure that all their subdivisions (e.g., counties) would resolve existing claims and bar future ones against the four companies [22]. All counties needed to make the decision on whether to sign on the settlement agreement or to continue pursuing lawsuits on their own within 180 days (by January 17, 2022) [61]. Without all counties signing the agreement, the state would receive partial settlement funds following a predetermined sliding scale. Therefore, to receive the full settlement amount, the goal of the Pennsylvania Office of Attorney General (OAG) was to achieve the full agreement from all counties [23].

The question that the OAG faced was how to distribute the settlement funds—the 70% of the total $1.07 billion—in “fair shares” that align with the communities’ needs in a consistent way across all counties. At the subdivision level, each county could view the allocation plan proposed by the OAG as a reasonable resolution and agree to the joint settlement agreement, or could pursue the lawsuit on their own that could result in potentially larger rewards subject to a higher risk and lengthy legal process. While larger counties may have more resources to pursue lawsuits on their own, there would be high uncertainty in the settlement amount even if they could reach a settlement on their own. Even if they would eventually win their lawsuits, many years would likely pass before any funds would be received. Local counties would prefer to receive funds sooner rather than later to address their imminent needs from the ongoing opioid crisis. Furthermore, not all counties are fully aware of the challenges and risks of pursuing the lawsuit on their own. As a result, the state government’s concern about counties not accepting the settlement is not negligible. In addition to the communication efforts of the OAG office with local communities to explain the benefit and importance of having full participation of all counties in the state [63], it is essential to present counties with a settlement distribution solution that is systematically developed from objective data to justify the needs of all counties.

### A formula-based solution

The OAG involved a community stakeholder group that included public health officials and attorneys representing local counties to determine the settlement allocation. The goal was to create an *evidence-based, transparent,* and *fair* distribution of the settlement funds deemed acceptable by all counties and stakeholders. The allocation needed to reflect the relative magnitude of the severity and negative consequences of opioid misuse in each subdivision. The analytical task was then reduced to identifying appropriate empirical metrics that were suitable to quantify the needs of communities and guide the allocation to support the needs of communities by providing resources for prevention, treatment, and harm reduction programs. A wide range of empirical metrics were identified from publicly available sources (for transparency and reproducibility) and were then assessed for their representativeness, relevance, and overall data quality with the feedback from the stakeholder group and external domain experts. Further discussion on the conceptual framework is provided elsewhere [23]. In summary, the final recommended allocation formula consisted of the following four metrics with different weights (we defer more detailed descriptions of data sources, selection criteria, and data limitations to Section 5.1):

- Number of overdose deaths from all drugs in 2015-2019 (40% weight)
- Number of individuals hospitalized for any opioid use disorder (OUD)-related disease in 2016-2019 (20% weight)
- Number of naloxone doses administered by Emergency Medical Services in 2018-2020 (20% weight)
- Intensity-adjusted amount of morphine milligram equivalents (MMEs) of prescription opioids dispensed in 2006-2014 (20% weight)

For each county, the percentage of the settlement allocated is calculated as the weighted sum of the percentage of each empirical metric for that county in the entire state. An additional consideration is the substantial urban/rural diversity of the counties in Pennsylvania. This matters because implementing abatement activities could be more challenging in rural areas given a more dispersed population and less developed health infrastructures to address the needs of behavioral health, compared with urban areas where scaling up abatement activities may be relatively easier under existing systems. To ensure that small counties received a meaningful amount of settlement funds for implementing abatement activities, Pennsylvania applied a *top-up minimum allocation* to its formula-based approach guaranteeing no county received less than $1 million in total [23]. That is, for counties with less than $1 million according to the formula-based allocation, their allocation would increase to $1 million while adjusting the remaining state allocation proportionally for the remaining counties above the minimum allocation.

Ultimately, all 67 counties within the Commonwealth agreed to the proposed formula-based allocation policy using a weighted sum of shares according to the above four empirical metrics [64]. While this agreement implies a level of acceptability in the Pennsylvania strategy that was developed as a quick, yet practical solution given a short response time, we remain interested in how the allocation decision question can be formulated differently to address the fairness of the settlement allocation in a more systematic manner.

### Can we do better?

It should first be recognized that Pennsylvania’s allocation solution is both transparent and intuitive, which also provided a timely solution that was deemed acceptable for all 67 counties. On the other hand, it does not necessarily represent the only solution as a gold standard approach to such a class of problems for the opioid settlement allocation—the 2021 settlement is not the first nor will it be the last. The underlying decision question is still worth revisiting to explore alternative solution approaches from different perspectives, to gain additional insights that could be used to support future allocation decisions in similar settings.

Specifically, we remark on two main areas where the current formula-based allocation may have limitations and could potentially be improved. First, although the ultimate goal of determining the allocation strategy is to achieve the *fairness* of the solution, the fairness outcome has not been objectively quantified. The current approach aimed to achieve fairness *implicitly* by soliciting and integrating feedback from community stakeholders until reaching a consensus. Through this decision-making process, fairness is used as a subjective assessment which could be perceived in different ways by different counties and stakeholders and may lack consistency and standard. Setting a minimum allocation amount is another strategy for promoting equity with good intentions, but its implications on fairness may still be viewed differently by stakeholders depending on their subjective opinions. A more *explicit* approach to addressing fairness using quantitative measures could further facilitate the deliberation throughout the allocation decision-making process.

Second, in the formula-based allocation, empirical evidence is synthesized following a simplistic linear form by weighting, where the weights represent the relative importance between the empirical metrics and were determined primarily based on expert opinions. Either by directly soliciting input for reasonable weights or using a more sophisticated approach to eliciting preferences such as the analytic hierarchy process, the values of these weights do not automatically account for fairness. It is also up for debate whether all counties should have the same weights for different metrics, considering that data quality and representativeness may vary across counties. Some counties may argue for a higher importance of certain variables over others. Acknowledging that a weighted sum formula is an acceptable and intuitive allocation solution, it may also be of interest for decision makers to explore potential further improvement if not confined to such a specific functional form for synthesizing empirical data.

With these observations, we are motivated to propose new mathematical formulations and optimization models that aim to address the fairness of the allocation by filling the above gaps in the next section. We do not do so as an afterthought—Pennsylvania’s model could still see potential use in future settlements and outlays. More importantly, our proposed approach has also laid an important guiding principle of an evidence-based approach that bases the allocation decision on a set of opioid-related population health outcome measures. For the interest of this study, we consider the set of empirical metrics as predetermined through external review and selection process, and focus on developing the method of utilizing these data to inform the allocation decisions, which could also see general use in other settings for distributing funds across subdivisions like counties or municipalities based on empirical data.

## Model formulations

In this section, we present the model formulations for the settlement allocation problem (SAP) with different definitions of fairness. We begin by introducing the notation and the generic optimization model. Then we discuss the rationale for defining fairness measures and introduce three fairness measures in the context of the opioid settlement allocation problem. Lastly, we introduce additional interpretability constraints to ensure that the allocation results follow an easy-to-interpret weighted-sum formula.

Consider a set of *N* counties (or non-overlapping communities in general) indexed by $i\in [N]$. We denote [*n*] as the set $\{1,\cdots, n\}$ for any given integer $n\in \mathbb {N}$. Let the decision variable $x_i$ for $i\in [N]$ represent the *percentage* of the total settlement that county *i* receives in the allocation. We refer to a solution of $\varvec{x}=(x_1, \cdots, x_N)'$ as an *allocation policy*.

Allocation policy $\varvec{x}$ will be determined based on a given set of *M* empirical measures. These empirical metrics characterize the burden of the opioid epidemic in the community (e.g., the four empirical metrics introduced in Section 3.1). Specifically, we denote $a_{im}$ as the value of measure $m\in [M]$ for county $i\in [N]$. Without loss of generality, we assume the values of $\varvec{a}=(a_{im})$ are scale-free, that is, representing only the *percentage* of the measured outcome that a given county contributes among all counties in the state, i.e., $\sum _i a_{im} =1$ for all $m\in [M]$. In addition, we define $a_i^{\min }$ and $a_i^{\max }$ as the lowest and highest metric values, respectively, for county *i*.

We take the perspective of a central decision maker (e.g., state attorney general) to optimize the allocation of the settlement dollars to all counties. The objective is to maximize the fairness of the allocation, ensuring that the allocated shares can justify each community’s needs and align with their needs consistently across all counties. In other words, the central decision maker wants to present all counties with a systematically developed allocation plan, where ideally no county would see their allocated share as being an unfairly low proportion relative to their needs compared to other counties. To model this, we first denote $F(\varvec{x},\varvec{a})$ as a generic *allocation fairness*, jointly determined by the allocation policy $\varvec{x}$ and the given empirical measures $\varvec{a}$. Before we elaborate with specific definitions of $F(\varvec{x},\varvec{a})$, we introduce the generic framework of optimizing the fairness of settlement allocation as follows:

1 $$\begin{aligned}&(\text {SAP})\quad \min _{\varvec{x}} \quad \left\{ F(\varvec{x},\varvec{a}) \Big| \sum _{i\in [N]} x_i = 1; \;\right. \nonumber \\&\qquad \qquad \qquad \left. a_i^\text {min} \le x_i \le a_i^\text {max}, \; \forall i\in [N] \right\} \end{aligned}$$

The first constraint in formulation Eq. 1 ensures that 100% of the available funds are allocated. The second constraint ensures that each county *i*’s allocation falls within the range of all empirical metrics $[a_i^{\max }, a_i^{\min }]$; allocation outside of this range, which is disproportionately high or low with respect to the empirical ranges, will be difficult to justify as an evidence-based solution and thus may be deemed unreasonable or unfair to other counties.

It is worth highlighting the major distinction of our SAP model Eq. 1 from the earlier-described formula-based approach (Section 3.1). The formula-based approach requires predetermined weights to differentiate the relative importance of different empirical measures, which has an intuitive structure for the allocation policy, but determining the values of these weights in consensus could be nontrivial. Instead, our SAP model adopts a data-driven approach, which views all available empirical measures as equally important and objective input data, to guide allocation decisions by defining a reasonable range for the allocated share, without weighing the measures based on subjective opinions.

### Remark 1

Our formulation takes a social planner’s viewpoint and focuses on maximizing the fairness of the allocation policy as a one-time decision. The underlying assumption is that all counties will be convinced to sign the joint agreement if the decision maker proposes a solution in good faith that can justify the needs of all counties in a relatively consistent way. We do not explicitly model an individual county’s signing decisions in response to a given allocation policy for several reasons. First, each individual county is weighing between a deterministic amount from the joint settlement and uncertain future rewards from their own litigation that is subject to risk of failure. The reward amount, timeline, and success probability in the latter case would all be very difficult to estimate. Second, there also exist complex political dynamics among the counties and between counties and the state that would be challenging to model. It may be of interest from a theoretical perspective to investigate these interactions and dynamics among multiple counties in their decisions using a game-theoretical framework. However, this is out of the scope of our study. Further, determining equilibrium in a game does not guarantee acceptability among stakeholders or a solution that cannot be improved [65].

### Allocation fairness

While it is generally agreed that fairness is an important consideration for resource allocation, there is no agreement on its definition. There is a vast economics literature, particularly in health economics, on the notion of distributional equity that concerns the fair distribution of goods or services [66]. The general notion of equity implies equality in the distribution of “something,” which is called the *focal point* by [67]. Many different focal points, such as access, utilization, expenditure, resources, needs, and health outcomes, are proposed in health care settings. Among these, three broad equity principles for guiding resource allocations have been commonly discussed with sustained attention: (1) allocation to ensure equality of access, (2) allocation according to need, and (3) allocation to produce an equal distribution of health [67, 68].

We argue that *allocation according to need* is deemed a more suitable principle in the specific context of the opioid settlement allocation. First, we should be reminded that settlement funds allocated to each county can only be spent on a prescribed set of abatement activities, such as treatment and harm reduction interventions, which aim to minimize the negative impact of the opioid crisis. It implies that the allocated resources are not intended to improve health access of the *general population*, such as access to primary care and preventive services; instead, the resources are targeted to improve access to substance use treatment and services for the *population at risk* of substance misuse and overdose. How different needs are for these at-risk populations across different counties should be understood first. We also do not follow the *equality of health* as the guiding principle for allocating the opioid settlement, because interventions and services for substance use are usually complex and it could take a long time to assess their impact on health outcomes, which could also be affected by an individual’s behavioral factors, social determinants of health, and other external factors. It is different from the situations of allocating food, drinking water, flu vaccines, and some medications that can typically yield immediate effects on quickly measurable health outcomes.

Therefore, we follow the equity principle of allocation according to each region’s relative need for substance treatment and services to further develop our fairness measures of settlement allocation. As an analogy to the example of using health expenditure of residents as the need for care in a region compared to other regions [66, 69], it is reasonable to use opioid-related outcome measures that are the results of the opioid epidemic to serve as the need of a region.

For the application of the opioid settlement allocation, however, the open question is how to synthesize multiple empirical measures to represent the relative need. Moreover, given the measures collected for different domains, it is impractical to unify the measuring units directly across all metrics. Therefore, instead of transforming multi-dimensional measures into a scalar variable representing the relative need and allocating resources proportionally accordingly, we employ a different solution strategy starting with defining the allocation fairness for any allocation policy, given a set of empirical metrics, and then optimizing the fairness measure. In the following sections, we introduce formulations and discuss the rationales of two intuitive allocation fairness measures based on the application context and one utility-based fairness measure established in the literature.

#### Minimizing deviation

Our first definition of allocation fairness is based on how closely the allocated shares match the empirical metrics, which we refer to as *deviation.* The rationale for such a definition is that a fair share of allocation should be aligned with the data of the empirical metrics in the same county. Multiple metrics, in scale-free form, as presented in percentages, can be viewed as different observations of the relative needs of each county. Then the goal of the central decision maker is to make each county’s share as close to the observations as possible, i.e., to minimize the overall deviations. Using the *absolute* differences as $(x_i - a_{im})$ could be problematic because for small and rural counties, their values of $a_{im}$ are usually much smaller than those for large urban counties by several orders of magnitude, and thus their deviations will be effectively overlooked. As a result, the allocation policy may be primarily driven by only a few large counties. Therefore, we instead use the *relative* differences with respect to the values of the empirical measures to define the *deviation*, $F_d(\varvec{x},\varvec{a})$, as an allocation fairness measure, which calculates the sum of squared relative differences by multiple empirical metrics and then takes the average across all counties:

2 $$\begin{aligned} F_d(\varvec{x},\varvec{a}):= \frac{1}{N}\sum _{i\in [N]} \sum _{m\in [M]} \left( 1-\frac{x_i}{a_{im}}\right) ^2. \end{aligned}$$

We call the solution to SAP Eq. 1 under the allocation fairness definition $F_d$ in Eq. 2 as *min-deviation allocation* policy. The values of empirical metrics $\varvec{a}$ in the definition $F_d$ are assumed to be strictly positive for notation simplicity. In practice, it is possible that one county has zero occurrences of a specific outcome; in that case, we can skip the relative deviation term $(1-x_i/a_{im})$ for the specific metric and county in the formula Eq. 2 and compute the average instead of the summation accordingly.

The SAP problem with allocation fairness $F_d$ can be viewed similarly to the least squares estimation problem, which treats the empirical metrics $\varvec{a}$ as the observed data points of unknown variables $\varvec{x}$ and aims to generate the best estimator that minimizes the total errors weighted by $1/a_{im}$ at each data point (in addition to the unity and range constraints in Eq. 1 for $\varvec{x}$). It is easy to see that the optimization problem Eq. 1 with $F_d$ is a convex quadratic program, for which a global optimal solution exists and can be efficiently solved numerically.

##### Remark 2

(L1-norm) The definition of $F_d(\varvec{x},\varvec{a})$ in Eq. 2 applies the L2-norm to the deviation terms to impose more penalties on outliers. Technically, it is also feasible to use the L1-norm instead, which can be easily linearized. The optimization problem then becomes a linear program as a result.

##### Remark 3

(Connection with simple average) One can consider a different scaling factor when calculating the relative deviation. If the deviation is scaled by the average of multiple metrics from a county (i.e., $\bar{a}_i:=\frac{1}{M}\sum _m a_{im}$) or by the same scalar within a county, the optimal allocation will be $x_i^* = \bar{a}_i$ immediately. This can be easily shown by checking the optimality conditions and verifying the feasibility constraints.

#### Minimizing maximum regret

While the allocated settlement funds are used in part to remedy past harms from the opioid crisis, there is no standard way to specify which empirical metric is the most representative of the harm. Each county can argue that the metric in its favor is more important than others and thus should be used as the standard. The *ideal* scenario where no county would complain is when all counties receive their allocation following $a_i^{\max }$, the highest possible share among the given set of empirical metrics, which is impossible simply because $\sum _i a_i^{\max }>1$. It is natural for counties to advocate for the use of their most *favorable* metric if their allocated amount is much lower, which is indeed a part of conversations with local stakeholders during the process of developing the formula-based allocation in Pennsylvania.

We denote the gap between the allocated amount $x_i$ and the county’s best possible amount $a_i^{\max }$ as the *regret* of the county. For a given allocation policy $\varvec{x}$, some counties may have low (or even zero) regret, but some may have a high regret value. A county with a high regret may feel being treated unfairly, especially when compared with other counties with much lower regret, and thus may refuse to sign the settlement agreement, risking the possibility of resolution. Since the goal of the central decision maker is to secure the participation from *all* counties, the decision question is reduced to how to make all counties less “unhappy” (with a lower regret); more importantly, it is critical to make the county with the largest regret to be “least unhappy” to minimize the risk that this county refuses to cooperate. Following the above rationales, we take a “worst-case” perspective and define an alternative allocation fairness measure $F_r(\varvec{x},\varvec{a})$ as the *maximum regret*:

3 $$\begin{aligned} F_r(\varvec{x},\varvec{a}):= \max _i \left( 1-\frac{x_i}{a_i^{\max }}\right). \end{aligned}$$

With this allocation fairness measure, the SAP problem Eq. 1 minimizes the maximum regret across all counties, and we call the optimal solution to this formulation as the *minimax-regret allocation* policy. Such a formulation also tends to reduce the range of regret across the parties involved. Due to similar concerns about drastically different orders of magnitude of $a_{im}$ across counties when defining Eq. 2, we also use the *relative* gap to define the regret, instead of directly computing the absolute gap $(a_{i}^{\max }-x_i)$. We also remark that there is no need to consider squared regret in definition Eq. 3 (similar to using L2-norm in Eq. 2), as the solution achieving the highest regret immediately maximizes the squared regret and vice versa. Then SAP problem Eq. 1 can be reformulated as a linear program:

4 $$\begin{aligned}&\min _{\varvec{x}, v} \left\{ v \Big \vert v\ge 1-\frac{x_i}{a_i^{\max }},\forall i\in [N]; \right. \nonumber \\&\quad \left. \sum _i x_i = 1; x_i \in [a_i^\text {min}, a_i^\text {max}], \forall i\in [N] \right\}. \end{aligned}$$

#### Maximizing alpha fairness

In addition to the above two fairness measures derived from the context of opioid settlement allocation, we also consider general fairness definitions from the existing literature [21] that are constructed based on utility values and social welfare functions (SWF). In our analysis, we choose *alpha fairness*, a measure capturing the trade-offs between equity (the worst-case reward) and efficiency (the total reward) to connect our proposed allocation fairness metrics specific to the settlement allocation problem setting with the generic fairness metrics in the literature, which could help us better situate our approach in the related area. We do not use fairness measures that solely quantify *inequality* of utilities across entities (e.g., Gini index, Hoover index), as achieving equal distribution of utilities across counties is not the goal for fair allocation of opioid settlement, nor a sufficient proposition to convince all counties to sign the agreement. Fairness prioritizing the disadvantaged group can be more relevant in the context of opioid settlement allocation, which is captured by the alpha fairness criteria.

The alpha fairness is defined as a class of SWFs in the following form, given the utility value $u_i$ of each county *i*,

5 $$\begin{aligned} W_\alpha (\varvec{u})={\left\{ \begin{array}{ll} \frac{1}{1-\alpha } \sum _i u_i^{1-\alpha }& \text {if } \alpha \ge 0, \alpha \ne 1, \\ \sum _i \log (u_i) & \text {if } \alpha = 1. \end{array}\right. } \end{aligned}$$

The alpha fairness measure is regulated by the parameter $\alpha$, which quantifies the priority given to the least-advantaged group. That is, when $\alpha \rightarrow \infty$, the alpha fairness reduces to the maximin criteria that emphasizes the least-advantaged group; when $\alpha =0$, it reduces to the utilitarian criteria by maximizing total utility (efficiency). In other words, the higher the value $\alpha$ is, the greater emphasis alpha fairness places on equity in terms of the utility values distributed across subgroups. The special case when $\alpha =1$ is well known as the *proportional fairness* measure. The solution that maximizes the proportional fairness is called the *Nash bargaining solution* [47], the equilibrium of the Nash bargaining game, which has also been commonly used in resource allocation problems in a variety of engineering settings [70, 71]. To define the utility $\varvec{u}=(u_1,\cdots, u_N)'$ of all counties for any given allocation policy $\varvec{x}$, we follow a commonly used approach called proportional scoring [72, 73]:

6 $$\begin{aligned} u_i = \frac{x_i - a_i^{\min }}{a_i^{\max }-a_i^{\min }},\;\forall i\in [N], \end{aligned}$$

which *scales* the allocated share $x_i$ to county *i* linearly within the range of the empirical data in this county. This way, each county has a utility ranging between 0 and 1. Then the fairness measure to be minimized in the SAP problem Eq. 1 is given as

7 $$\begin{aligned} F_\alpha (\varvec{x}, \varvec{a}):= - W_\alpha \left( \varvec{u}(\varvec{x},\varvec{a})\right), \end{aligned}$$

where the SWF $W_\alpha$ is defined in Eq. 5 and the utility function $\varvec{u}$ in Eq. 6.

Although the resulting formulation is nonlinear, it is convex for all $\alpha \ge 0$. Thus, the SAP with alpha fairness measure remains a convex optimization problem, which is numerically tractable and guarantees global optimality.

### Interpretable allocation

Although the simple formula-based allocation policy, used as a practical solution to allocate the opioid settlement in Pennsylvania (Section 3), does not explicitly quantify fairness, it has its own merits in clearly and transparently communicating to stakeholders how the final allocation is determined from empirical data, making it easy for policymakers to interpret and explain the allocation approach. This advantage motivates us to account for such a simple structure in our formulation.

If we take the allocation policy $\varvec{x}^*$ directly from the above optimization formulations to “reverse-engineer” the corresponding weights $\varvec{w}=(w_1, \cdots, w_M)'$ by solving a system of linear equations $\varvec{A}\varvec{w} = \varvec{x}^*$ where matrix $\varvec{A}=[a_{im}]$, the linear system may not be feasible since it is likely that $N\gg M$ (e.g., $M=4$ metrics used to inform allocation across $N=67$ counties in Pennsylvania).

Instead, we enforce the weighted sum representation of the allocation policy $\varvec{x}$ directly in the SAP formulation Eq. 1 by adding the following constraints about weights $\varvec{w}$ and allocation $\varvec{x}$:

8a $$\begin{aligned}&\sum _{m\in [M]} a_{im} w_m = x_i,\quad \forall i\in [N], \end{aligned}$$

8b $$\begin{aligned}&\sum _{m\in [M]} w_m = 1, \end{aligned}$$

8c $$\begin{aligned}&w_m \in [w^{\min }, w^{\max }], \quad \forall m\in [M], \end{aligned}$$

where $w^{\min }$ and $w^{\max }$ respectively represent the lower and upper limits for weights $\varvec{w}$. Policymakers can choose acceptable limits other than the natural bounds of 0 and 1 to avoid overly unbalanced weights across empirical measures, such as one measure dominating the others. Following the above definition, the OAG’s formula-based solution can be viewed as a feasible solution in the form of Constraints Eq. 8 with $x_i=\varvec{a}_i'\varvec{w} =$ (share of drug overdose deaths) $\times$ 0.4 + (share of OUD-related hospitalizations) $\times$ 0.2 + (share of naloxone administered by EMS) $\times$ 0.2 + (share of prescription opioids dispensed) $\times$ 0.2 for county *i*.

## Computational study

In this section, we present our numerical results for the opioid settlement allocation policies based on empirical data for Pennsylvania [23]. Throughout these results, we aim to understand how allocation policies with different fairness formulations impact the allocated share for a county and their underlying reasons. We will also discuss the disparities in allocation fairness by factors that are not explicitly accounted for in the fair allocation formulation. That is, how different is the fairness of allocation achieved in some counties (e.g., rural, or with lower median household income) compared to others (e.g., urban, with higher median household income)? How are such disparities differed by allocation policies? Lastly, we will evaluate the price of interpretability by examining how much it will impact the fairness measures and the resulting disparities, if the allocation policies must conform to the simplistic and intuitive form as the weighted sum of the given set of empirical metrics.

### Empirical metrics and data sources

For each opioid-related empirical metric for Pennsylvania, we compute the share of each county ($a_{im}$) as the percentage of the total number of events in that county over the selected year range among the grand total of all counties in the state. These empirical metrics are obtained from various public data sources for transparency and reproducibility. The final set of metrics and year ranges are selected based on availability, relevance, representativeness, and overall data quality [23] (see [74–76] for broader discussions on opioid and substance use-related data issues). In the following, we summarize the data sources, selection criteria, and rationale for each of these metrics.

#### Drug overdose deaths

The number of overdose deaths is the prominent measure of the opioid crisis, representing the most devastating outcome to both patients and society. This metric has been widely used as the outcome measure in extensive health policy literature to understand the distribution patterns of the opioid crisis across demographics, geographics, and time [77–80], and to evaluate the impact of intervention policies [81–84]. Following a well-documented approach in the literature [81, 84] that utilizes the Centers for Disease Control and Prevention’s Wide-ranging Online Data for Epidemiologic Research (WONDER) Multiple Cause of Deaths database [85], we obtain the number of overdose deaths from all drugs during the years 2015-2019 (with underlying cause of death ICD-10: X40-44, X60-64, X85, Y10-14; and multiple cause of death ICD-10: T36-50) for each county. We include overdose deaths from all drugs, rather than those from opioids only for two reasons. First, emerging data have shown that the opioid epidemic has been shifting to polysubstance use patterns, particularly including the use of stimulants [3, 86]. Being inclusive of the types of drugs involved in overdose deaths and focusing on the most recent 5-year window with available data (at the time of the analysis) would be more appropriate to capture the fast-growing and rapidly evolving nature of the opioid crisis, which better reflect the current and future abatement needs. Second, it has been argued that overdose deaths from opioids have been under-reported in WONDER [87]. It is known that the capability of toxicology testing at the coroner’s office to determine the exact types of drugs that caused deaths may also vary substantially across counties. In fact, we indeed observe the case where overdose deaths from opioids specifically are disproportionately underestimated for some counties of Pennsylvania, implying that the opioid overdose death data may not be adequately representative at the county level. Three rural counties (Cameron, Forest, and Sullivan) have suppressed values (when the actual value was below 10), and we replace suppressed values with 9s as a conservative assumption for these counties, which is expected to result in a very limited impact on the overall distribution across other counties.

#### Naloxone administrations

In addition to overdose deaths, non-fatal overdoses could also represent the burden of opioid use in communities, which can partially be captured by naloxone administration. We compute the number of naloxone units administered in each county from the incident-level records of naloxone administered by Emergency Medical Services (EMS) based on reports completed by certified EMS providers in the National EMS Information System (NEMSIS) [88]. Records with the Provided Primary Impression of “Overdose/Poisoning/Ingestion” and provider-administered naloxone or naloxone hydrochloride are included. Data from January 2018 through October 2021 are available at the time of the analysis and so we select the records for three full years of 2018-2020. We remark that there are two potential limitations of this dataset. First, the county information in this dataset is determined by incident location, rather than the patient’s residence. Second, in practice, not all naloxone is administered by EMS, as it may also be given by law enforcement. In this dataset, the observed naloxone administrations may be skewed towards higher values in urban areas due to better coverage of EMS, whereas rural areas may be more likely to be underestimated due to limited access to EMS and inadequate coverage.

#### Opioid use disorder (OUD)-related hospitalizations

The numbers of unique individuals hospitalized for any OUD-related diseases by each county during years 2016-2019 (latest available as of the time of data extraction) are estimated from Pennsylvania Health Care Cost Containment Council (PHC4) [89]. As an independent state agency, PHC4 collects more than 1.7 million inpatient hospital discharge records from hospitals in Pennsylvania each year, including detailed clinical and administrative data, which are systematically collected across the state, and thus could comprehensively represent the distribution across counties. The estimates for OUD-related hospitalization include all hospitalization records that had an OUD diagnosis code and the primary diagnosis with any one of OUD, intracranial and intraspinal abscess, endocarditis, osteomyelitis, endocarditis, soft skin tissue infection, or viral hepatitis (B, C, and D), which serves as a surrogate metric of county-level OUD prevalence to represent the geographical distribution of the disease burden. The county information in this dataset is determined based on the patient’s residence, which could more accurately represent the geographic distribution of disease burden rather than the location of admitting hospitals. Counts of OUD-related hospitalizations below 11 were all suppressed in the public file, which we impute by the value of 10 as a conservative estimate. Imputation was primarily made for small and rural counties, and the total sum of the imputed values constituted <2% of the total number of hospitalizations. These minor overestimates for small and rural counties were unlikely to substantially affect the overall distribution of the measure, and their impact on the settlement allocation for large and urban counties was minimal.

#### Opioids dispensed

The amount of prescription opioids dispensed characterizes the supply of opioids in the community that has fueled the opioid epidemic [90]. The total amount of dispensation by county, measured in morphine milligram equivalents (MME), is obtained from the Automation of Reports and Consolidated Orders System (ARCOS) data [91]. The data are only available for the years 2006-2014 at the time of the analysis. The ARCOS data collect comprehensive transactional records of all controlled substances from manufacturers and distributors to the Drug Enforcement Administration (DEA) [92]. To derive the county-level estimates, we follow the same criteria as documented in the Expert Report provided for the court [61] in the national opioid litigation by including (1) transactions for sales only, (2) 12 types of prescription opioids excluding buprenorphine and methadone that are typically used for OUD treatment, and (3) transaction with buyer type of dispenser only (excluding manufacturers, distributors, reverse distributors, researchers, labs, importers, and exporters). It is argued that not all dispensed opioids were misused in the same way across all counties, and thus the relative scales of the damage to the communities could not be simply captured by the amount of opioids dispensed. To account for this, we follow a similar adjustment approach used in the settlement negotiation with minor modifications due to data availability (see details in Appendix A).

### Settlement allocation policies under different fairness measures

Using the empirical metrics calculated from the above data sources, we solve the optimization models with different allocation fairness criteria of min-deviation, minimax-regret, and $\alpha$-fairness with $\alpha$ varying over a wide range of values $\{0, 0.5, 1, 2, 5, 10.95, 15\}$. In addition to the known special cases of $\alpha =0$ (i.e., the utilitarian criteria) and $\alpha =1$ (i.e., the proportional fairness yielding the Nash bargaining solution), we consider reference values $5-10.95$ for $\alpha$ based on the range of empirical estimates of the “inequality aversion” parameter in Atkinson Index across socioeconomic groups [50, 51]; $\alpha =15$ is sufficiently large and increasing it further will not result in meaningful numerical differences in allocation policies, which can then be viewed close to the maximin criteria with $\alpha =\infty$. All optimization models (including quadratic and logarithm function constraints) are solved to optimality using Gurobi 11.0.

#### Comparison of allocation policies

**Fig. 1:**
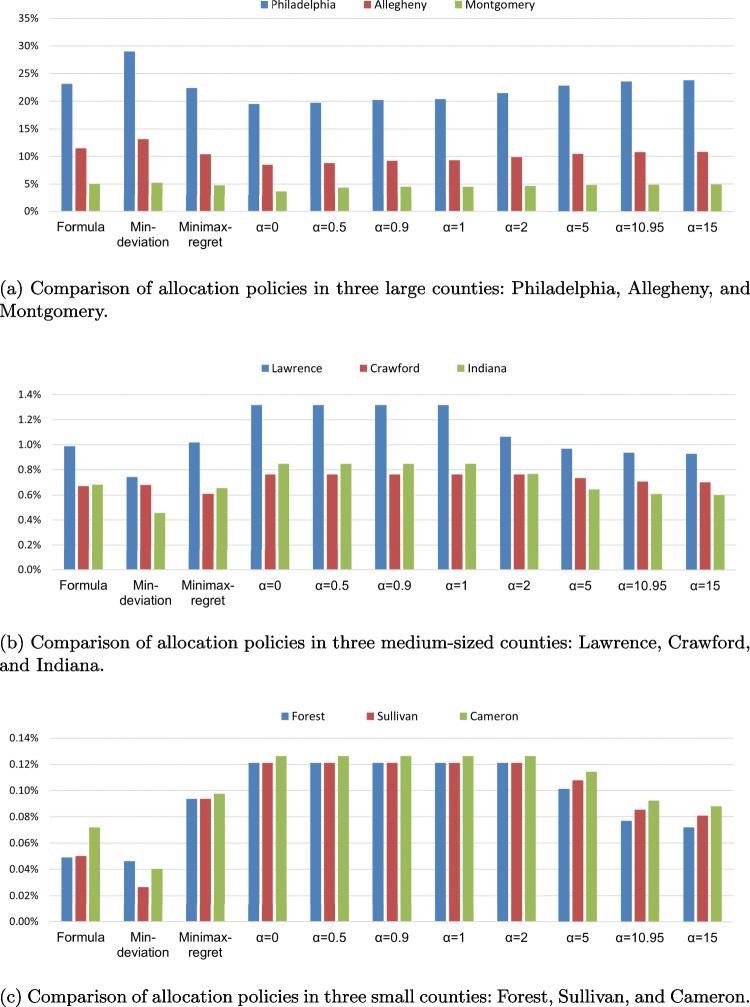
Comparison of settlement allocation policies in three example counties of (A) large, (B) medium, and (C) small sizes

All allocation policies for each of the 67 counties in Pennsylvania are provided in Appendix Table B1. As expected, given the large variations in the counties’ sizes, the allocated amounts differ significantly by several orders of magnitude over a wide range. For example, the allocated percentage of the total settlement can be as high as 10%-30% for large counties like Philadelphia and Allegheny Counties, and as low as <0.1% for small and rural counties like Sullivan and Cameron Counties. Considering the formula-based allocation as a baseline for comparison, we also observe variations of the allocated shares by the optimal allocation policies under different fairness metrics surrounding the baseline results. For ease of discussion about the impact of allocation policy on these diverse counties in different ways, we select three counties with the highest (Philadelphia, Allegheny, and Montgomery), median (Lawrence, Crawford, and Indiana), and lowest population sizes (Forest, Sullivan, and Cameron) to represent large and urban, mid-size, and small and rural communities, respectively, which makes the allocation results more comparable within each of these groups (Fig. 1).

We first find that the min-deviation allocation tends to favor large counties. As shown in Fig. 1, compared with other allocation policies, the min-deviation allocation gives the highest percentages to the three largest counties while the lowest to small counties. Such a discrepancy is mainly driven by how much the empirical metrics vary relatively in these counties. For example, in a large county like Philadelphia, the values of empirical metrics range between 19%-29%, implying that for any given allocated percentage within this range, the relative deviation cannot be lower than -34% (=(19%-29%)/29%) or above 53% (=(29%-19%)/19%); however, for a small county like Sullivan with the empirical metrics ranging between 0.02%-0.12%, where the upper limit of the range is 6 times of the lower limit, the relative deviation of a given allocation could be anywhere from -83% to 500%! In fact, it is very common that in small counties, the metrics are typically positively skewed, with most metrics concentrated on the lower side of the range while having a metric with a large value isolated from the rest. Because relative deviation $\vert x_i-a_{im}\vert / a_{im}$ is more sensitive for a small value $a_{im}$ by definition (i.e,. given the same absolute deviation $\vert x_i -a_{im}\vert$, the relative deviation will be much higher for small $a_{im}$), when minimizing the overall deviation, the allocated percentages in small counties “gravitate” towards the lower side of the feasible range to avoid the high penalty due to deviating from the low metric values too far. Thus, the min-deviation allocation may penalize the counties more when they have large variations in the empirical metric values and most metrics have very low values, which is quite common for small and rural counties.

In contrast to min-deviation allocation, minimax-regret allocation tends to distribute the settlement more favorably to smaller counties. For example, the allocated share increases by more than one-third in mid-size counties like Indiana and Lawrence, and nearly doubled in all three counties in the small size group (Fig. 1). With the optimal allocation policy under the minimax-regret criterion, the maximum regret is 22.7%, meaning that the actual allocated amount is 22.7% lower than the most favorable metric value in that county. In fact, out of 67 counties in total, only 2 counties attain a lower regret, only because these two counties’ maximum possible regret $(a_i^{\max }-a_i^{\min })/a_i^{\max }$ is less than 22.7% due to small variations in their empirical metrics. In other words, in the minimax-regret allocation policy, all counties, *whenever possible*, receive the lowest possible allocation as long as their regret is no worse than anyone else. When following the min-deviation criterion, allocations to small counties tend to be drawn closer to lower metric values and thus result in a higher regret; when switching to minimax-regret criterion, minimizing the largest regret can be viewed as a process of rebalancing the large regrets in small counties and small regrets in large counties. By giving out only a small amount of the settlement money, several large counties’ regret will not increase too much, but the amount re-distributed to many small counties can substantially decrease their regret, especially given the multiple order-of-magnitude lower values $a_i^{\max }$ in these small counties. Therefore, it makes intuitive sense to expect higher allocation to smaller counties in the minimax-regret allocation policy as we observe in our results. Appendix Fig. B1 further illustrates the preference of the minimax-regret allocation policy towards small counties, whereas the min-deviation policy towards large counties.

For $\alpha$-fairness allocation policies, as $\alpha$ increases (i.e., the allocation leans more heavily on equity over efficiency), large counties tend to receive more allocation while small counties tend to receive less. Such differences can be explained by the observation that redistributing a small amount of allocation from large to small counties will only marginally decrease the utility of large counties in exchange for substantially increased utility in small counties. This is because the empirical metrics tend to be more dispersed over a wider range in large counties and more concentrated at small values within a narrow range in small counties. Given that the utility is inversely proportional to the range $a_i^{\max }-a_i^{\min }$ in Eq. 6, the same redistributed amount could result in larger gains of the utility in small counties than the losses in large counties. Therefore, maximizing the total utility when $\alpha =0$ results in a strong incentive to reallocate shares from large to small counties, as a result, to favor small over large counties. Conversely, when $\alpha$ is large, the decision maker has less incentive to do so, as it may not necessarily improve the new objective that no longer maximizes the total utility. The decision maker is also less motivated to increase the allocated shares in small counties—as long as they are not the most disadvantaged counties—under the new objective, resulting in lower allocations in these counties, as observed in Fig. 1.

**Fig. 2:**
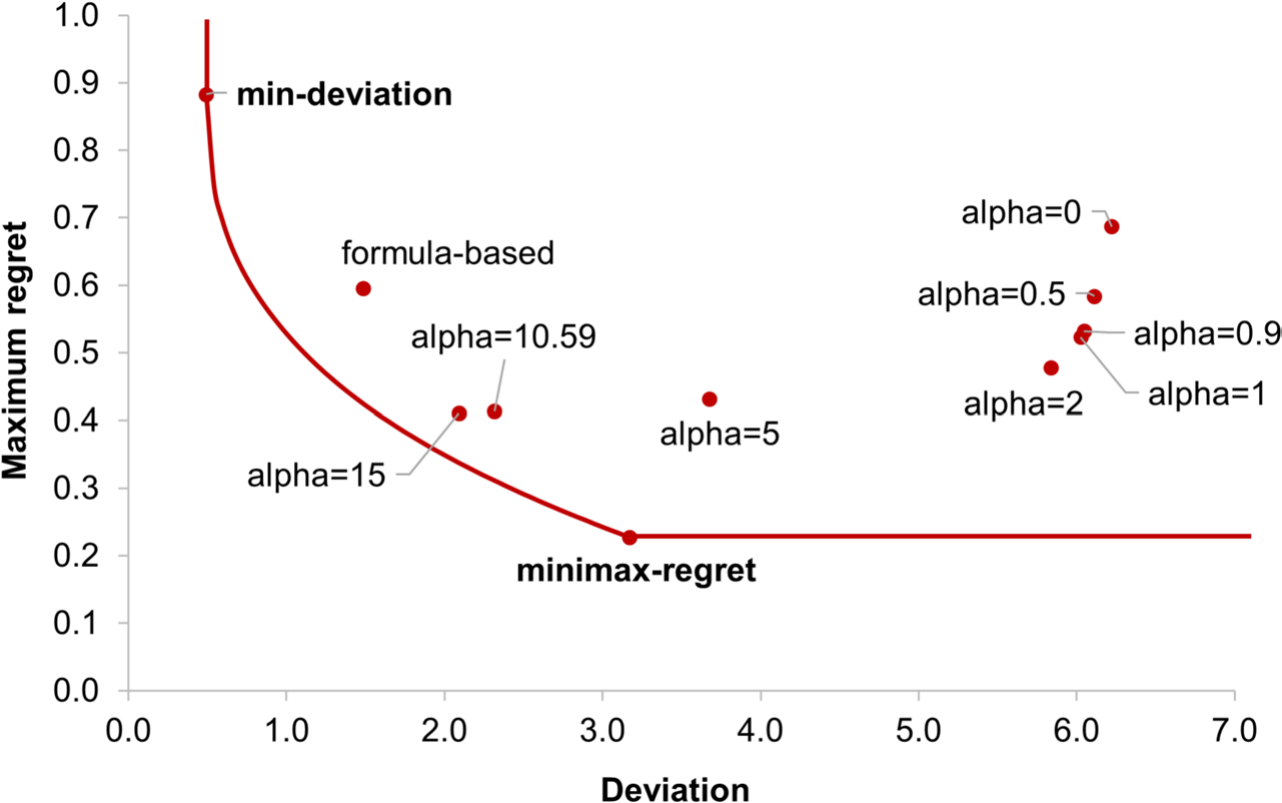
Deviation-regret frontier and comparison of multiple allocation policies by the two allocation fairness criteria. The frontier is calculated based on a sequence of upper bounds $\epsilon$ for the deviation with an increment value of 0.05 and between the deviation of the min-deviation and minimax-regret allocation policies

#### Deviation-regret frontier

To explore the trade-offs between the two fairness measures, namely, deviation and maximum regret, and to understand the gap of other allocation policies to achieve the best possible fairness outcomes, we employ the multiobjective optimization framework [93] to generate a Pareto frontier consisting of a set of non-dominated allocation solutions with respect to the two fairness measures, which we refer to as the *deviation-regret frontier* in our problem setting. Specifically, we apply the $\epsilon$-constraint method, a widely used technique of generating the Pareto frontier [94] that has several advantages over other techniques like the weighting method [95], by optimizing one fairness measure (e.g., the maximum regret $F_r(\varvec{x},\varvec{a})$) while bounding the other (e.g., deviation $F_d(\varvec{x},\varvec{a})$) by a series of $\epsilon$ values. That is, the deviation-regret frontier is constructed by repeatedly solving the following optimization problem,

$$\begin{aligned}&\min \left\{ F_r(\varvec{x},\varvec{a}) \Big \vert F_d(\varvec{x},\varvec{a})\le \epsilon; \right. \\&\qquad \left. \sum _i x_i =1; x_i \in [a_i^{\min },a_i^{\max }],\forall i\in [N] \right\}, \end{aligned}$$

with a sequence of values for the bound $\epsilon$ with an increment of 0.05 in the range $[D^{\min },D^{\max }]$, where $D^{\min }$ and $D^{\max }$ are attained by the min-deviation and minimax-regret allocation policy, respectively. For any $\epsilon$ value below the deviation $F_d(\varvec{x},\varvec{a})$ at the min-deviation allocation, the problem becomes infeasible.

The deviation-regret frontier and fairness measures of all allocation policies are depicted in Fig. 2. The bottom-left direction in this figure indicates a better fairness outcome of an allocation policy and the upper-right region of the frontier represents the feasible region. Any policies lying in this region are dominated by the solutions on the frontier. The deviation-regret frontier shows the trade-offs between the two fairness metrics. In other words, the frontier shows the boundary at which one fairness measure cannot be further improved without compromising the other (Fig. 2). That is, there exists no other allocation policy that can possibly improve both fairness measures at the same time. As $\alpha$ increases, the $\alpha$-fairness allocation policies move towards the bottom-left direction and are approaching the frontier; the cases $\alpha =10.59$ and $\alpha =15$ are the closest to the frontier. An interesting observation is that the formula-based allocation used in the Pennsylvania opioid settlement is also very close to the frontier, compared with most other $\alpha$-fairness allocation policies, implying a reasonable balance between the two allocation fairness measures by the intuitive structure of this simple formula.

### Disparities in settlement allocation

A given finite set of empirical metrics may not capture all the differences between counties that could have influenced the settlement distribution. Other qualitative features of counties, such as rural-urban areas, economic well-being, and existing healthcare infrastructures, could also be important considerations for policymakers to distribute the settlement funds across counties, but these features have not yet been explicitly considered in the previous analysis. Such a gap naturally leads to a question about potential disparity in the fairness of the settlement allocation, that is, whether or to what extent a certain group of counties could receive a systematically less equitable allocation compared with the rest? Understanding the disparity could provide an additional view of the overall fairness of the settlement allocation from a dimension outside of the empirical metrics, especially when comparing multiple non-dominated allocation policies that are on or close to the frontier as shown in Fig. 2.

**Fig. 3:**
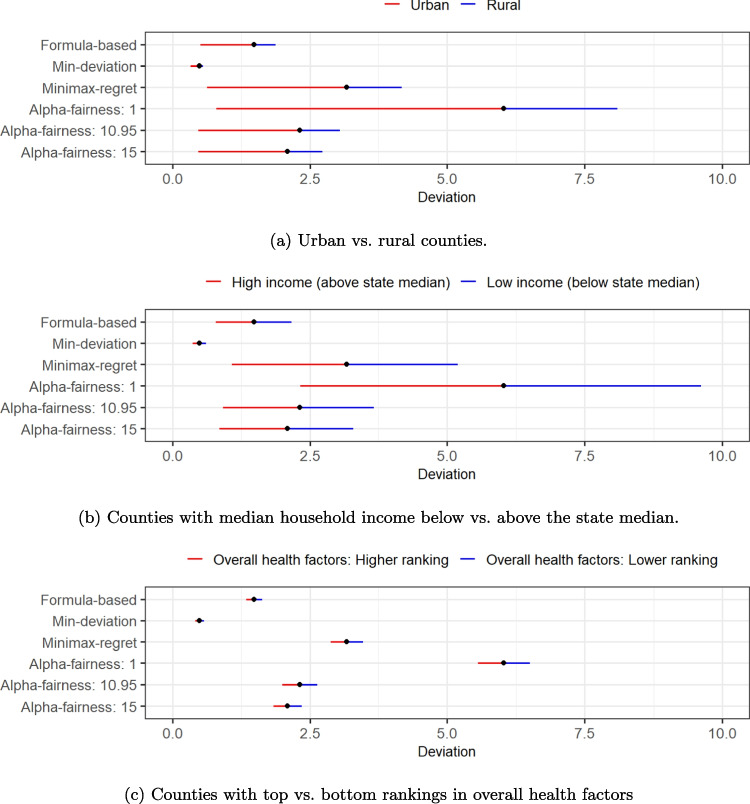
Disparity in the allocation fairness measured by deviation between (a) urban vs. rural counties, (b) counties with median household income below vs. above the state median, and (c) counties with top vs. bottom rankings in overall health factors

For ease of interpretation and discussion, we evaluate and compare the allocation fairness between dichotomous subgroups of counties. Specifically, we partition all counties by (1) rural and urban areas, which is one of the most prominent distinctions for counties geographically as they oftentimes represent substantial differences in resource availability and face different challenges, (2) median household income above or below the state median level, a crucial economic indicator that helps gauge the economic well-being of the population in a region, and (3) health factors from the published County Health Rankings & Roadmap [96], which have systematically assessed and synthesized a wide range of social determinants of health and provided relative rankings for all counties; in particular, we dichotomize the counties by the top and bottom halves in the ranking of overall health factors. We then evaluate the allocation fairness measures, deviation and maximum regret, for each partition of counties separately (Fig. 3).

We first focus on the disparity in the relative deviation between urban and rural counties. Figure 3a compares the deviation for rural counties (blue line), urban counties (red line), and all counties (black point) for each allocation policy. The right-end line segment represents a higher deviation and thus poorer fairness and the left-end line segment for the opposite, and the total range represents the disparity. Across all allocation policies, rural counties consistently have higher deviations compared with urban counties, implying that allocations to rural counties deviate more from the empirical metrics than urban counties. One possible reason is that rural counties tend to have higher variations and a wider range across the empirical metrics in their relative scales with sparse and sporadic observations, which makes the allocation more difficult to match with the values of empirical metrics. Among the allocation policies, min-deviation allocation shows the lowest disparity between rural and urban counties. When counties are partitioned by other factors, we observe that counties with lower median household income (Fig. 3b) and those with lower-ranking health factors (Fig. 3c) have higher deviations and poorer fairness of allocation. Min-deviation allocation consistently shows the lowest disparity compared with other allocation policies.

Disparities in maximum regret show a similar pattern where rural, lower household income, and worse health factors have higher maximum regret for the allocation, except for a few cases where comparative scales are reversed by only a small amount (Appendix Fig. B2). Across different allocation policies, while $\alpha$-fairness allocations with large $\alpha$ values tend to have lower disparities, the minimax-regret allocations consistently show zero disparity regardless of how counties are divided, because it minimizes disparity by design: The formulation of minimax-regret allocation suppresses all counties’ regret from above uniformly and results in the same regret for all counties whenever this regret is achievable. Additionally, we plot the deviation versus regret under different allocation policies for all counties color-coded by subgroup in Appendix Fig. B3. The patterns in these scatter plots consistently show the disparities and poorer fairness outcomes across the allocation policies for counties in rural areas, with lower household income, and with lower-ranking health factors, compared with their counterparts.

### Price of interpretability

**Fig. 4:**
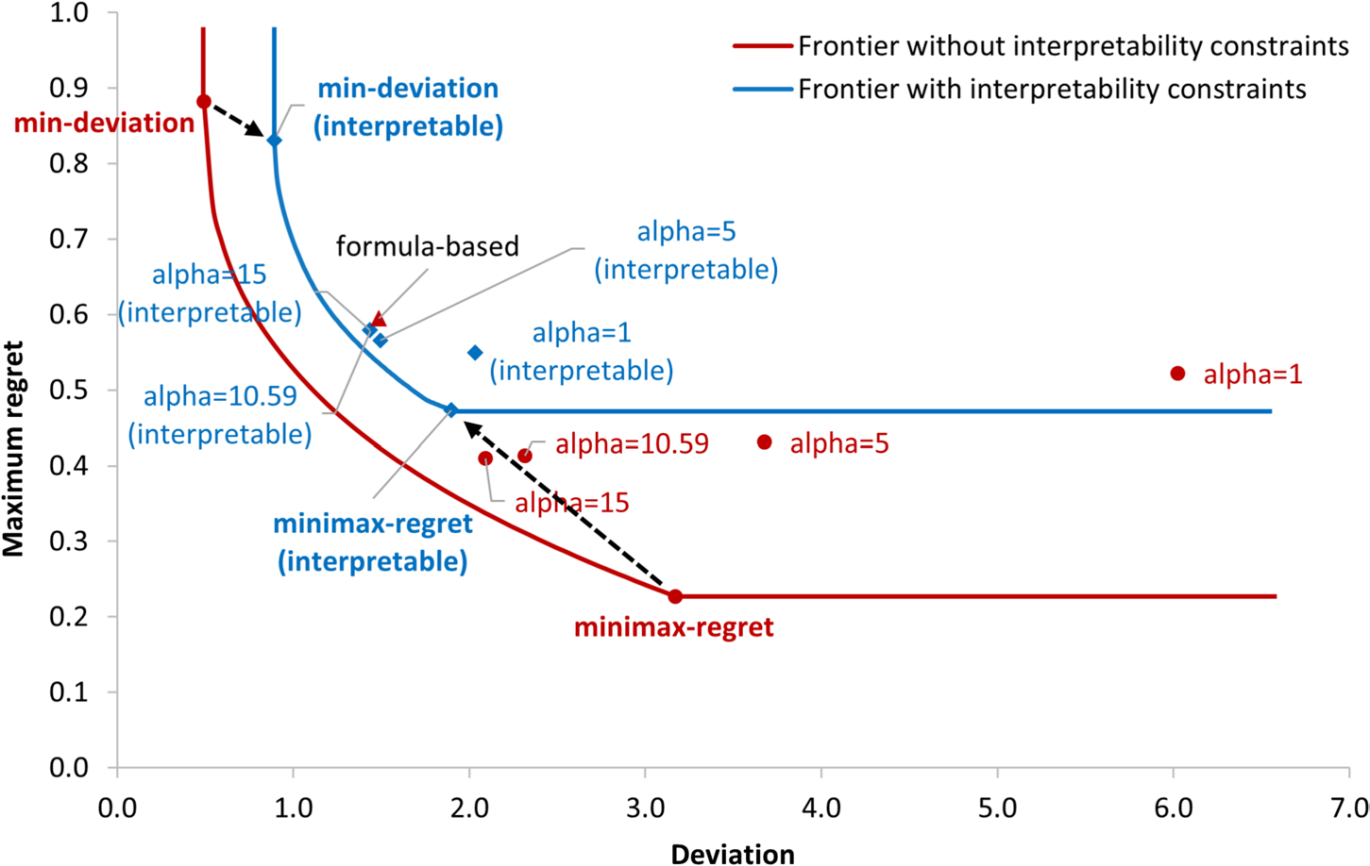
Comparison of deviation-regret frontiers and allocation policies with interpretability constraints. The frontier is calculated based on a sequence of upper bounds $\epsilon$ for the deviation with an increment value of 0.05 and between the deviation of the min-deviation and minimax-regret allocation policies with or without interpretability constraints, respectively

The allocation policies calculated from the different optimization models are purely data-driven, which may lack interpretable structures that could be crucial to support the policymakers’ decision process. To examine the trade-off between fairness and interpretability, we restrict the allocation policies to be the weighted sum of the given set of empirical metrics, following the form of the formula-based approach employed in the settlement allocation in Pennsylvania (Section 3) as formulated in Section 4.2, allowing the model to determine the optimal weights (Appendix Table B2). For practical purposes, we limit the weight of each empirical metric below 0.9 to prevent one single metric from dictating the allocation policy. We then compare interpretable allocation policies with the original ones (Fig. 4) and discuss the impact of adding the interpretability constraint.

For the min-deviation allocation, adding the interpretability constraint increases the deviation from 0.49 to 0.89, an 82% increase (Fig. 4). The increase in the deviation, implying a decrease in allocation fairness, is interpreted as the *price of interpretability*. The interpretability constraint does not necessarily penalize the other fairness measure, maximum regret, of the min-deviation allocation policy, as it is not explicitly considered in the objective of the optimization; in fact, the maximum regret of min-deviation allocation decreases slightly when the interpretability constraint is added, as an unintended benefit. Similarly, for the minimax-regret allocation, adding the interpretability constraint doubled the maximum regret from 0.23 to 0.47, while its deviation is reduced substantially. All $\alpha$-fairness allocations also show increases in their maximum regret as the price of interpretability. We re-create the deviation-regret frontier for interpretable allocation policies (the blue line in Fig. 4), and observe that the frontier shifts “behind” the one without the interpretability constraint as expected, as no interpretable allocation policy can outperform the original frontier. With the interpretability constraint, $\alpha$-fairness allocations also show a consistent pattern of approaching the frontier as $\alpha$ increases. Figure 4 also shows that most shifting of the equity measures is along the direction of increasing maximum regret, implying that this equity measure is deemed more sensitive to the interpretability constraints.

The disparity in allocation fairness may also be impacted by the interpretability constraint. For min-deviation allocation, adding interpretability constraints leads to higher deviations in both rural and urban counties, while enlarging the disparity between them at the same time (Appendix Fig. B4). However, other allocation policies show lower deviations and smaller disparities between rural and urban counties, compared with the allocations without considering interpretability constraints. These results imply that the prices of interpretability in terms of the deviation can impact both the average and variability of allocation fairness consistently. For the fairness measure of maximum regret (Appendix Fig. B5), the interpretability constraint increases the disparity between rural and urban counties in most cases and has the least impact on the disparity of the minimax-regret allocation policy. Similar patterns can also be observed when counties are grouped by median household income and overall health factors.

## Discussion and conclusions

A fair allocation should ensure that all groups are relatively well provided with resources according to their unique needs. We define two allocation fairness measures for this specific problem context, *deviation* and *maximum regret*, and optimize the allocation based on each. We show the deviation-regret frontier to demonstrate the trade-off between the two fairness measures. The frontier also represents the boundary for any potential improvement of allocation fairness, which could provide policymakers with useful references to gauge how far a given allocation policy is from achieving the best possible fairness outcomes when evaluating a broader set of alternative allocation policies. We also compare the allocation policies based on alpha fairness with varying $\alpha$ values and found that the alpha fairness allocation approaches the frontier as $\alpha$ increases (away from the purely utilitarian allocation with $\alpha$=0 and towards equitable allocation). Moreover, it is important to understand potential disparities that can arise from the chosen allocation method. We compare the fairness measures of the various allocation policies between counties in rural versus urban areas, with high median income versus low median income, and having a high versus low health factor ranking. It is of interest that the min-deviation allocation has the lowest deviation and the least difference in the deviation measure, and hence leads to the smallest disparities. The other allocation strategies lead to higher deviations for rural, low-income, and low-health-ranked counties. The alpha fairness ($\alpha$=1) allocation has the greatest disparities in each case, although the disparities are reduced as $\alpha$ increases.

We note that the two fairness measures developed in this work—deviation (representing average alignment between empirical data and the allocated shares) and maximum regret (representing the worst case)—differ from existing fairness measures in the utility and social welfare function framework. This is due to the specific problem context of opioid settlement allocation. In particular, the objective of the allocation is to have buy-in from all counties. In addition, the settlement agreement stipulates that the funds must be used for abatement strategies for remediating the harms of the opioid crisis. How much a given amount of settlement funds maps to each county’s utility, however, is not readily defined, and also depends on how each county chooses to invest available funds over a wide range of intervention programs. Instead, we measure the past damages incurred by opioid deaths, the number of individuals hospitalized for any OUD-related disease, the number of naloxone doses administered by EMS, and the amount of prescription opioids dispensed, use them as proxies to quantify the needs of each county, and define our fairness measure around how differently these needs are aligned with the allocated shares across counties. In our case study based on real-world empirical data of Pennsylvania, allocations that are closer to the min-deviation fairness measure tend to favor larger counties, while those closer to the minimax-regret measure tend to favor smaller counties.

Interpretability of the allocation can be an important factor in terms of transparency when discussing the allocation results with stakeholders such as local government officials and representatives. A benefit of the formula-based allocation, a weighted sum of several empirical metrics, is its simplicity and easy interpretability. To account for such an interpretable structure, we introduce additional constraints that restrict each of the allocation strategies to the functional form of a weighted sum in the optimization formulation. The interpretability comes with a “cost”: Each point on the interpretable deviation-regret frontier is further away from the origin than the original frontier. The cost is more significant in terms of maximum regret compared to deviation.

Another important distinction with the formula-based allocation is that the weights in the interpretable allocation formulation are not predetermined to evaluate the importance of the measures but are optimized for the given set of empirical measures to present how the allocation policy could be explained by these data. This provides better transparency to communicate with local communities as opposed to a black-box solution. These data-driven weights are likely to change for a different set of measures or in a different state. Moreover, this interpretable formulation can be easily extended to incorporate additional structures, such as preferred orders or ranges for the weights, to further facilitate communications with the local communities throughout the decision-making processes.

The formula-based allocation used by the state is dominated by other interpretable allocation policies, but does not lie far from the deviation-regret efficiency frontier, and is very close to the interpretable frontier. In addition, the disparities arising from the formula-based allocation are lower than the allocations based on minimax-regret and alpha fairness measures. Overall, accepting the virtues of simplicity and interpretability in the formula-based allocation strategy used by Pennsylvania, an optimization-based strategy could have provided a fair allocation policy that is more acceptable to more stakeholders. While in the case of our computational study, the min-deviation strategy performed very well when considering the interpretability constraint, we intend the allocation strategies presented here to provide a framework for facilitating future discussions of allocation decisions.

The optimization-based approach provides an alternative way of utilizing the empirical data. Unlike the formula-based approach that requires weighting the relative importance of different empirical metrics based on subjective opinions, the SAP model formulation does not distinguish between the importance of the included metrics and views all data as equally important and relevant input to guide the allocation decisions. In this way, it alleviates the burden for decision makers to reach a consensus about the relative importance of each metric among stakeholders.

Our analytical framework presented in this study serves as an initial step towards informing fair allocations of opioid settlements using a systematic and data-driven approach, which can be further extended to capture several additional considerations and complexities in real-world practice. For example, the settlement allocations may involve political subdivisions that are not entirely mutually exclusive, such as allocating resources among counties and municipalities (cities or townships) at the same time. In some states, there is also possible coordination between counties and cities. In Kentucky, where the original allocation plan results in a city receiving a total of less than $30,000 in any individual settlement, judgment, or bankruptcy proceeding, the payment is instead made to the county, consolidated local government, or urban county government in which that city is located. Certain situations could be more complex than what has been formulated in the model of this study, which would require ad-hoc modifications based on more operational details. Furthermore, we confine our analysis to the four specific metrics used in the formula-based allocation. This decision was due in part to the desire to include the formula-based allocation in Pennsylvania for comparison. These metrics are also all publicly available and hence support transparency. It is possible that if different measures were included, the results could differ. That said, the basic framework, namely the structure of the fairness measures, can be applied to other sets of measures used.

Our allocation model did not explicitly account for possible collaborations between counties to address the spatial spillover effects on the impact of the substance use epidemic and its damage to population health. In our analysis, we intend to minimize the impact of spillover effects by carefully choosing empirical metrics that are calculated based on patients’ residence locations whenever available, instead of using the locations of the treatment event or the treatment facility. On the other hand, in practice, services required for prevention, treatment, and harm remediation often occur and are coordinated in a group of subdivisions (e.g., counties), especially in small and rural areas, as joint efforts in combating this public health crisis, instead of being operated entirely independently between subdivisions. In Pennsylvania, 47 Single County Authorities (SCA) are formed for 67 counties across the commonwealth, overseen by the Pennsylvania Department of Drug and Alcohol Programs (DDAP), as the entity designated for planning, coordination, and management of the delivery of intervention and services at the local level specifically for drug and alcohol-related issues. It may also be a meaningful strategy for counties to consolidate resources and develop joint efforts, which can leverage existing infrastructure without duplicating the fixed costs of establishing new administrative or programmatic infrastructure in order to maximize the population health impact. An additional limitation is that we only know as a fact that the formula-based settlement allocation was accepted by all counties in Pennsylvania and cannot guarantee that our proposed allocation policies that dominate the formula-based allocation to be immediately accepted in practice. We believe the fairness measures and the analytical approach presented in this study will provide a practical framework to support and inform the stakeholders’ discussion and their decision process of developing an agreeable allocation policy.

After the state allocates lump-sum amounts of the opioid settlement to the local level, the next-level allocation decisions are left to local governments and agencies to choose intervention strategies. This leads to a new set of resource allocation problems that warrant systematic and evidence-based approaches to optimize the intervention strategies. As the epidemic continues to evolve, efforts are needed to closely monitor the emerging patterns of the epidemic, to track actual spending on the intervention programs, and to accumulate evidence to update the understanding of efficacy and cost-effectiveness of various intervention strategies in community settings, which will provide timely information to guide local decision makers towards more effective use of opioid settlement funds.

## Supplementary Information

Below is the link to the electronic supplementary material.

Supplementary file 1 (pdf 412 KB)

## Data Availability

All data supporting this study are obtained from publicly available sources, for which references are provided in the manuscript text. Analysis results are provided in this paper and its Supplement Materials.
