# Supplementary material for: Fair allocation strategies for opioid settlements
Source: Health Care Manag Sci. 2025 Aug 7;28(3):335–56. doi: 10.1007/s10729-025-09716-8 (PMC12535511; doi:10.1007/s10729-025-09716-8)
Supplement: Supplementary file 1 — (pdf 412 KB) [file 10729_2025_9716_MOESM1_ESM.pdf]

Electronic Supplementary Materials Accompanying "Fair Allocation Strategies for Opioid Settlements"

Appendix A Severity-based adjustment to the amount of opioid dispensed

The original adjusting multiplier was defined as follows:

$$\max\left\{\frac{\text{A county's OUD prevalence rate}}{\text{state level OUD prevalence rate}}, \frac{\text{A county's opioid overdose death rate}}{\text{state-level opioid overdose death rate}}\right\},$$

which means that, intuitively, if the county has a higher than state-average prevalence rate (per 10,000 population) or a higher overdose death rate (per 10,000 population), this severity level of this county is deemed higher and this county has the adjusting multiplier value >1.0.

However, because the county-level prevalence rate and opioid overdose death rate with Ruhm adjustment [1, 2] used in the original calculation were not based on publicly available data, the prescribed approach could be reproduced exactly. Instead, we calculated the OUD-related hospitalization rate (defined as [average annual number of hospitalizations for OUD-related diseases during 2016-2019]/[county population]*100,000) as the proxy for the OUD prevalence rate. One potential issue of this method is that for rural counties where we replaced the suppressed hospitalization counts by value 10, it resulted in exceptionally high rate when divided by a small population size. In such cases, to ensure reasonable value for the adjusting multiplier, we upper bounded the ratios in these counties by the ratio in Philadelphia County which had the highest value among all counties without suppressed counts. Similarly, we used the all-drug overdose death rate (defined as [average annual number of all drug overdose deaths 2015-2019]/[county population] *100,000) to replace the opioid overdose death rate in the severity adjusting factor definition.

Appendix B Additional results of fair allocation policies

Table B1: Solutions of fair settlement allocation policies.

County	Formula-based	Min-deviation	Minmax-regret	Alpha = 0	Alpha = 1	Alpha = 5	Alpha = 10.59	Alpha =15
Adams	0.344%	0.310%	0.361%	0.467%	0.467%	0.410%	0.379%	0.372%
Allegheny	11.524%	13.132%	10.434%	8.500%	9.334%	10.488%	10.758%	10.818%
Armstrong	0.606%	0.405%	0.560%	0.725%	0.725%	0.562%	0.525%	0.517%
Beaver	1.274%	0.886%	1.341%	1.735%	1.511%	1.251%	1.228%	1.221%
Bedford	0.192%	0.158%	0.200%	0.258%	0.258%	0.238%	0.213%	0.208%
Berks	1.891%	1.508%	1.855%	2.400%	1.884%	1.747%	1.737%	1.733%
Blair	0.807%	0.791%	0.713%	0.922%	0.922%	0.838%	0.804%	0.797%
Bradford	0.225%	0.163%	0.238%	0.308%	0.308%	0.266%	0.237%	0.231%
Bucks	5.803%	6.473%	5.729%	4.969%	5.803%	6.090%	6.147%	6.158%
Butler	1.369%	1.242%	1.268%	1.640%	1.640%	1.384%	1.348%	1.340%
Cambria	1.587%	0.888%	2.332%	1.452%	1.440%	1.715%	1.769%	1.779%
Cameron	0.072%	0.040%	0.098%	0.126%	0.126%	0.114%	0.092%	0.088%
Carbon	0.644%	0.601%	0.756%	0.978%	0.978%	0.812%	0.776%	0.768%
Centre	0.250%	0.169%	0.252%	0.326%	0.326%	0.275%	0.245%	0.238%
Chester	2.230%	1.612%	2.272%	2.940%	1.997%	2.032%	2.045%	2.046%
Clarion	0.147%	0.111%	0.140%	0.181%	0.181%	0.167%	0.144%	0.139%
Clearfield	0.299%	0.142%	0.319%	0.412%	0.412%	0.314%	0.279%	0.272%
Clinton	0.105%	0.080%	0.103%	0.133%	0.133%	0.128%	0.109%	0.105%
Columbia	0.328%	0.289%	0.326%	0.421%	0.421%	0.378%	0.349%	0.343%
Crawford	0.671%	0.680%	0.610%	0.762%	0.762%	0.732%	0.705%	0.699%
Cumberland	1.048%	0.938%	0.963%	1.246%	1.246%	1.054%	1.018%	1.009%
Dauphin	1.611%	1.514%	1.509%	1.952%	1.952%	1.720%	1.685%	1.676%
Delaware	6.468%	6.615%	7.563%	4.700%	5.533%	6.715%	6.993%	7.055%
Elk	0.168%	0.096%	0.208%	0.270%	0.270%	0.219%	0.189%	0.183%
Erie	2.051%	2.129%	1.993%	2.579%	2.579%	2.258%	2.225%	2.217%
Fayette	1.348%	1.170%	1.326%	1.715%	1.655%	1.323%	1.294%	1.286%
Forest	0.049%	0.046%	0.094%	0.121%	0.121%	0.101%	0.077%	0.072%
Franklin	0.514%	0.352%	0.571%	0.739%	0.739%	0.560%	0.524%	0.515%
Fulton	0.077%	0.039%	0.094%	0.121%	0.121%	0.111%	0.089%	0.085%
Greene	0.205%	0.156%	0.228%	0.294%	0.294%	0.261%	0.234%	0.228%
Huntingdon	0.179%	0.129%	0.189%	0.245%	0.245%	0.214%	0.187%	0.182%
Indiana	0.683%	0.457%	0.655%	0.847%	0.847%	0.643%	0.607%	0.599%
Jefferson	0.189%	0.136%	0.193%	0.249%	0.249%	0.218%	0.191%	0.185%

Juniata	0.061%	0.036%	0.094%	0.121%	0.121%	0.109%	0.087%	0.083%
Lackawanna	1.393%	1.068%	1.351%	1.748%	1.651%	1.335%	1.308%	1.300%
Lancaster	2.266%	1.970%	2.090%	2.704%	2.266%	2.097%	2.083%	2.078%
Lawrence	0.989%	0.741%	1.018%	1.317%	1.317%	0.967%	0.935%	0.927%
Lebanon	0.603%	0.531%	0.799%	1.033%	1.033%	0.799%	0.763%	0.755%
Lehigh	2.107%	1.685%	2.454%	3.175%	1.980%	2.112%	2.141%	2.145%
Luzerne	3.282%	2.371%	3.754%	1.521%	2.355%	2.959%	3.084%	3.110%
Lycoming	0.642%	0.516%	0.849%	1.098%	1.098%	0.827%	0.792%	0.784%
McKean	0.180%	0.105%	0.194%	0.252%	0.252%	0.210%	0.181%	0.175%
Mercer	0.931%	0.766%	1.202%	1.555%	1.394%	1.106%	1.081%	1.074%
Mifflin	0.173%	0.119%	0.179%	0.231%	0.231%	0.203%	0.176%	0.171%
Monroe	0.827%	0.796%	0.759%	0.982%	0.982%	0.871%	0.836%	0.828%
Montgomery	5.047%	5.243%	4.780%	3.697%	4.530%	4.834%	4.894%	4.905%
Montour	0.143%	0.126%	0.156%	0.202%	0.202%	0.188%	0.166%	0.161%
Northampton	1.686%	1.416%	1.614%	2.088%	1.937%	1.645%	1.619%	1.612%
Northumberland	0.578%	0.540%	0.522%	0.675%	0.675%	0.621%	0.590%	0.583%
Perry	0.231%	0.166%	0.218%	0.282%	0.282%	0.246%	0.218%	0.212%
Philadelphia	23.146%	28.989%	22.403%	19.545%	20.379%	22.850%	23.568%	23.741%
Pike	0.283%	0.136%	0.315%	0.408%	0.408%	0.312%	0.277%	0.270%
Potter	0.067%	0.021%	0.094%	0.121%	0.121%	0.106%	0.083%	0.079%
Schuylkill	0.986%	0.965%	0.837%	1.078%	1.078%	1.013%	0.980%	0.974%
Snyder	0.109%	0.079%	0.109%	0.140%	0.140%	0.134%	0.115%	0.111%
Somerset	0.425%	0.318%	0.399%	0.516%	0.516%	0.428%	0.394%	0.386%
Sullivan	0.050%	0.027%	0.094%	0.121%	0.121%	0.108%	0.085%	0.081%
Susquehanna	0.166%	0.119%	0.172%	0.222%	0.222%	0.197%	0.172%	0.167%
Tioga	0.149%	0.135%	0.147%	0.191%	0.191%	0.183%	0.163%	0.159%
Union	0.082%	0.060%	0.094%	0.121%	0.121%	0.116%	0.098%	0.094%
Venango	0.336%	0.308%	0.419%	0.542%	0.542%	0.450%	0.416%	0.409%
Warren	0.139%	0.034%	0.225%	0.291%	0.291%	0.215%	0.182%	0.175%
Washington	1.647%	1.397%	1.565%	2.025%	1.915%	1.605%	1.578%	1.570%
Wayne	0.315%	0.183%	0.298%	0.385%	0.385%	0.310%	0.277%	0.270%
Westmoreland	3.227%	3.049%	2.853%	3.692%	3.031%	2.953%	2.950%	2.948%
Wyoming	0.204%	0.161%	0.210%	0.272%	0.272%	0.242%	0.215%	0.209%
York	2.571%	2.369%	2.253%	2.915%	2.483%	2.312%	2.297%	2.292%

Table B2: Weights of empirical metrics in interpretable allocation policies.

	**Weight of empirical metrics**			
**Allocation policy**	**Overdose deaths**	**OUD-related hospitalizations**	**Naloxone administrations**	**Opioids dispensed**
Formula-based	40.0%	20.0%	20.0%	20.0%
Min-deviation	8.1%	9.4%	68.9%	13.6%
Minimax-regret	7.5%	39.2%	19.5%	33.9%
Alpha fairness				
alpha=0	90.0%	10.0%	0.0%	0.0%
alpha=0.5	90.0%	10.0%	0.0%	0.0%
alpha=0.9	75.7%	21.4%	2.9%	0.0%
alpha=1	72.2%	22.9%	4.9%	0.0%
alpha=2	50.0%	27.6%	16.7%	5.7%
alpha=5	34.1%	26.6%	24.2%	15.1%
alpha=10.59	31.5%	25.6%	26.1%	16.9%
alpha=15	31.4%	25.6%	26.2%	16.8%

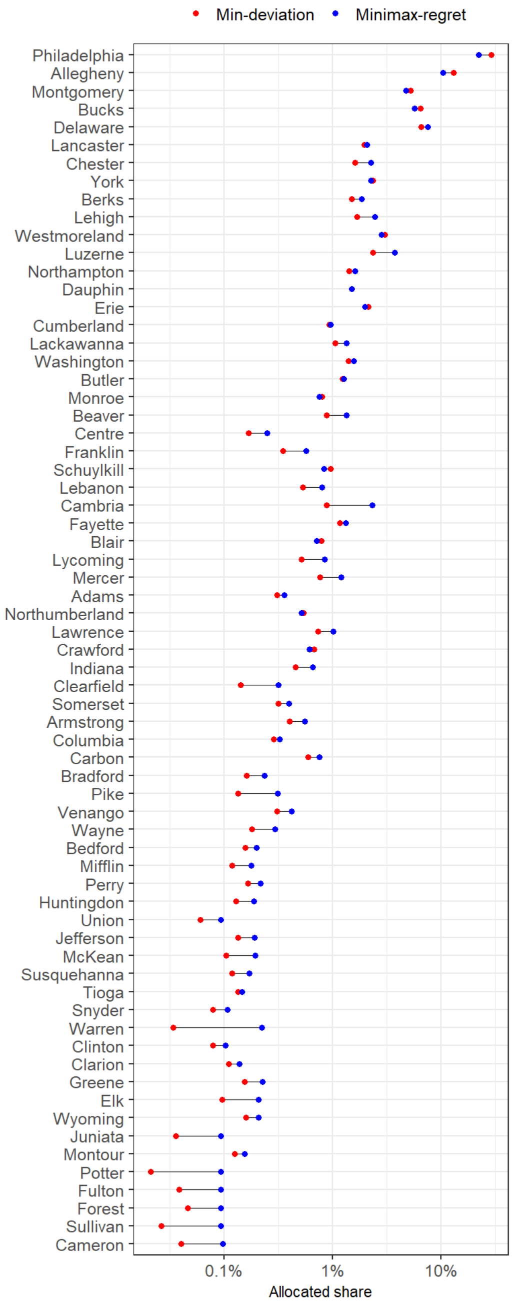

Fig. B1: Comparison of the allocated share of the settlement by each county between the min-deviation and minimax-regret allocation policies.

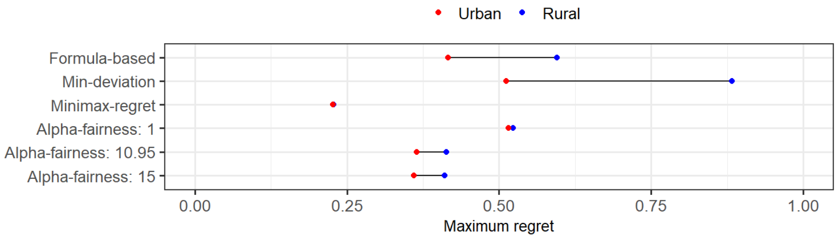

(a)

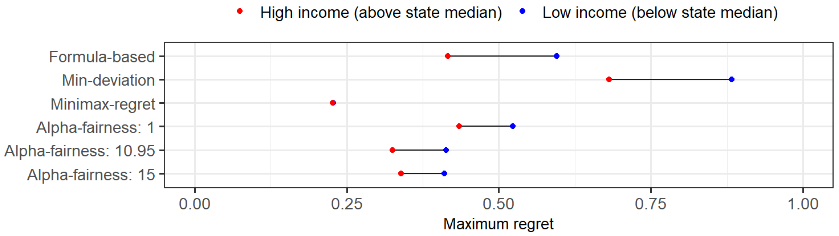

(b)

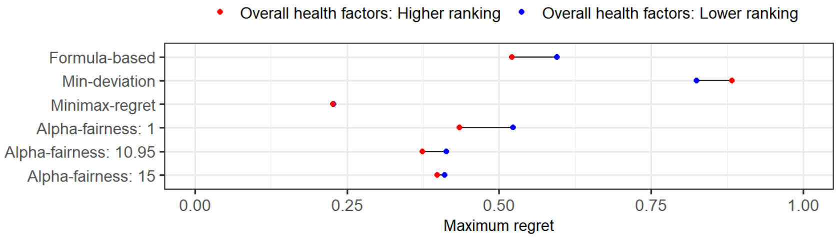

(c)

Fig. B2: Disparity in the allocation fairness measured by maximum regret between (a) rural vs. urban counties, (b) counties with median household income below vs. above the state median, and (c) counties with top vs. bottom rankings in overall health factors.

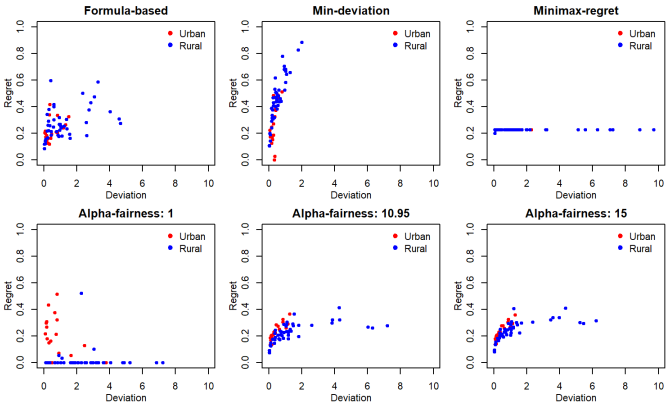

(a)

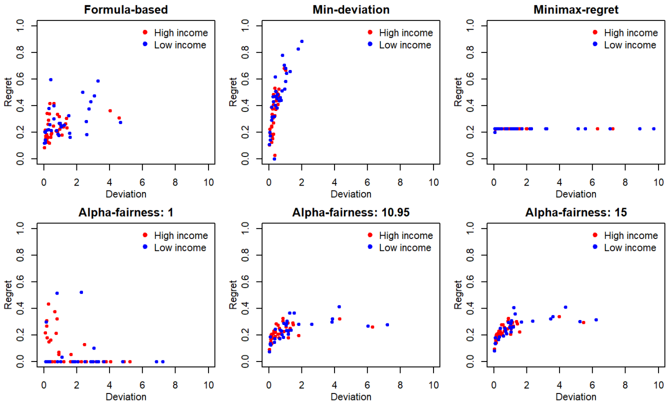

(b)

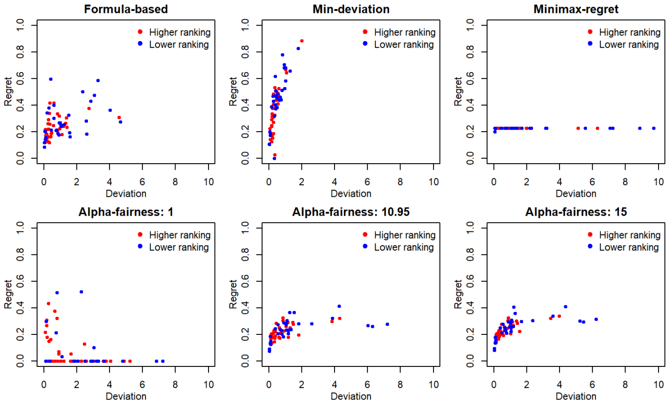

(c)

Fig. B3: Disparity in allocation fairness metrics of different allocation policies by (a) rural vs. urban counties, (b) counties with median household income below vs. above the state median, and (c) counties with top vs. bottom rankings in overall health factors.

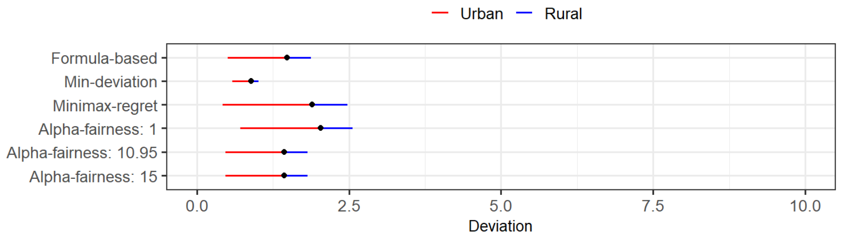

(a)

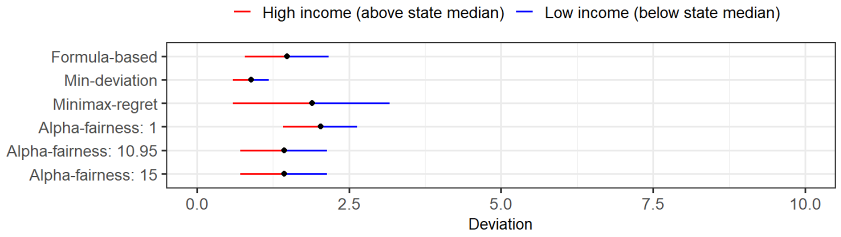

(b)

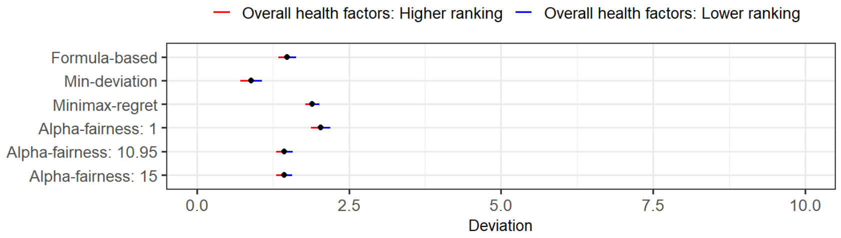

(c)

Fig. B4: Disparity in the fairness measured by deviation for allocation policies with interpretability constraint between (a) rural vs. urban counties, (b) counties with median household income below vs. above the state median, and (c) counties with top vs. bottom rankings in overall health factors.

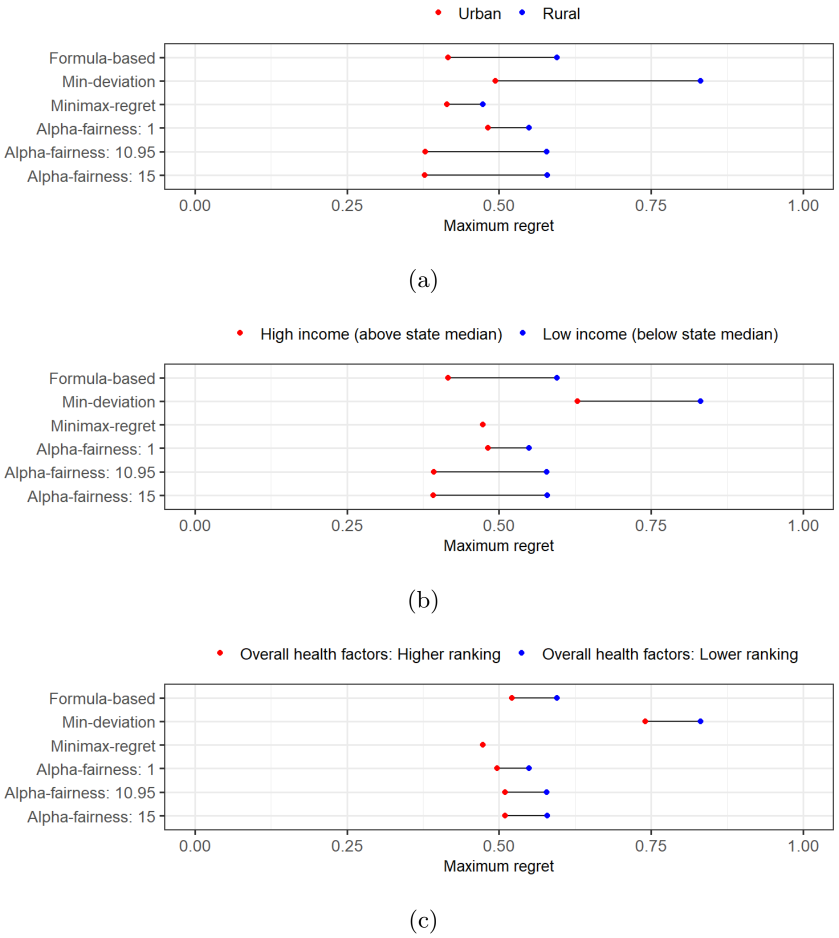

Fig. B5: Disparity in the fairness measured by maximum regret for allocation policies with interpretability constraint between (a) rural vs. urban counties, (b) counties with median household income below vs. above the state median, and (c) counties with top vs. bottom rankings in overall health factors.

References

[1] Ruhm CJ. Corrected US opioid-involved drug poisoning deaths and mortality rates, 1999–2015. Addiction. 2018;113(7):1339–1344.

[2] Ruhm CJ. Drug poisoning deaths in the United States, 1999–2012: a statistical adjustment analysis. Population health metrics. 2016;14(1):1–12.
